# Identification of the conserved long non-coding RNAs in myogenesis

**DOI:** 10.1186/s12864-021-07615-0

**Published:** 2021-05-10

**Authors:** Anupam Bhattacharya, Simang Champramary, Tanya Tripathi, Debajit Thakur, Ilya Ioshikhes, Satyendra Kumar Singh, Soumyadeep Nandi

**Affiliations:** 1 Division of Life Sciences, Institute of Advanced Study in Science and Technology, Vigyan Path, Paschim Boragaon, Garchuk, Guwahati, Assam India; 2 Department of Molecular Biology and Biotechnology, Cotton University, Panbazar, Guwahati, Assam India; 3 University of Szeged Faculty of Science and Informatics, Szeged, 6720 Hungary; 4 Functional Genomics and Bionformatics, University of Sopron, Sopron, Hungary; 5 Stem Cell & Cell Culture Lab, Centre For Advanced Research (CFAR), King George’s Medical University, Lucknow, UP India; 6 Ottawa Institute of Computational Biology and Bioinformatics (OICBB), Ottawa Institute of Systems Biology (OISB), Department of Biochemistry, Microbiology and Immunology (BMI),Faculty of Medicine, University of Ottawa, Ottawa, Canada; 7 Data Sciences and Computational Biology Centre, Amity Institute of Integrative Sciences and Health, Amity University Haryana, Gurugram, Manesar, 122413 Haryana India

## Abstract

**Background:**

Our understanding of genome regulation is ever-evolving with the continuous discovery of new modes of gene regulation, and transcriptomic studies of mammalian genomes have revealed the presence of a considerable population of non-coding RNA molecules among the transcripts expressed. One such non-coding RNA molecule is long non-coding RNA (lncRNA). However, the function of lncRNAs in gene regulation is not well understood; moreover, finding conserved lncRNA across species is a challenging task. Therefore, we propose a novel approach to identify conserved lncRNAs and functionally annotate these molecules.

**Results:**

In this study, we exploited existing myogenic transcriptome data and identified conserved lncRNAs in mice and humans. We identified the lncRNAs expressing differentially between the early and later stages of muscle development. Differential expression of these lncRNAs was confirmed experimentally in cultured mouse muscle C2C12 cells. We utilized the three-dimensional architecture of the genome and identified topologically associated domains for these lncRNAs. Additionally, we correlated the expression of genes in domains for functional annotation of these trans-lncRNAs in myogenesis. Using this approach, we identified conserved lncRNAs in myogenesis and functionally annotated them.

**Conclusions:**

With this novel approach, we identified the conserved lncRNAs in myogenesis in humans and mice and functionally annotated them. The method identified a large number of lncRNAs are involved in myogenesis. Further studies are required to investigate the reason for the conservation of the lncRNAs in human and mouse while their sequences are dissimilar. Our approach can be used to identify novel lncRNAs conserved in different species and functionally annotated them.

**Supplementary Information:**

The online version contains supplementary material available at 10.1186/s12864-021-07615-0.

## Background

Recent transcriptomic studies of mammalian genomes have revealed the presence of a substantial population of non-coding RNA (ncRNA) molecules among the transcripts expressed in cells. More than 90% of the human genome encodes ncRNAs [1–3], and the presence of such a large collection of ncRNAs indicates the regulatory potential of these molecules [4–6] . Based on size, ncRNAs are grouped into two classes: short ncRNAs and long ncRNAs. Short ncRNAs, fewer than 200 bp in length, include microRNAs or piwi-interacting RNAs; long ncRNAs (lncRNAs) are greater than 200 nucleotides and transcribed mostly by RNA polymerase II. Similar to messenger RNAs, lncRNAs contain a 5′7-methylguanosine cap and a 3′ poly(A) tail; however, lncRNAs lack coding potential. This new class of genes has recently been identified in various tissues [7–10]. Although the functions of microRNAs are well studied [11], the mode of action of lncRNAs in gene regulation is not well understood. Previous studies in X-chromosomal dosage compensation underscore the regulatory potential of lncRNAs, whereby the mechanism is carried out via concerted action of the lncRNA Xist and protein complexes [12, 13]. Recent studies have revealed the involvement of lncRNAs in Drosophila dosage compensation. This dosage compensation system employs two lncRNAs (roX1 and roX2), which are essential for other proteins to form the Male-Specific lethal complex and for targeting of the complex to hundreds of distinct sites on the X chromosome in male fruit flies [12, 14–17]. Recent studies provide evidence that lncRNAs play important roles in normal physiology and many diseases [6], including embryonic stem cell maintenance, differentiation and development [18], the antiviral response [19], gene imprinting [20], and cancer progression, as well as vernalization in plants [21]. Furthermore, the ENCODE project (GENCODE v26) has annotated thousands of lncRNAs in various cells [6], though further studies are required for functional annotation of these lncRNAs.

In addition, evidence for the involvement of lncRNAs in embryonic or adult skeletal myogenesis and muscle diseases is growing [22–27]. Therefore, we selected the process of myogenesis as a case study to identify lncRNAs from large transcriptome data in mice and humans and annotated the functional roles played by lncRNAs in skeletal myogenesis. We determined differentially expressed lncRNAs in myoblasts and myotubes and confirmed expression with epigenetic marks, such as histone modifications. Additionally, we determined conserved lncRNAs by investigating the shared synteny of the lncRNA with nearby genes in both mouse and human. We further functionally characterized the identified lncRNAs based on their association with the genes in their vicinity. In general, lack of sequence homology and conserved secondary structure of these lncRNAs make the functional annotation a challenging task [28–30], and there have been many previous attempts at functionally annotating lncRNAs. In some cases, the function of lncRNAs has been inferred by exploring relationships between lncRNAs and nearby protein-coding genes [31], and some roles have been predicted by identifying coding genes co-expressed with lncRNAs [32, 33]. We obtained the structures of the identified lncRNAs from Conserved-RNA Structure (CRS) database [34]. Some of these lncRNAs show moderate structural conservation, which also indicates a common role in mice and humans. Subsequently, we functionally characterized lncRNAs by examining the gene ontology of neighbouring genes, as well as by investigating the ontologies of genes in close vicinity in three-dimensional space. Some of these identified lncRNAs were experimentally validated in C2C12 cells, and the results revealed that the computationally identified lncRNAs are indeed differentially expressed in these cells.

## Results

The objective of this study was to identify conserved lncRNAs between humans and mice. Hence, we first identified lncRNAs present in mice and correlated their expression with nearby genes, epigenetic marks and histone modifications. The expression of a few identified lncRNAs was experimentally confirmed. The lncRNAs identified from mice were compared with human datasets to identify conserved RNAs. Finally, the functional role of these lncRNAs was assessed by overlapping them with topologically associated domains and investigating the function of the genes in these domains.

### Identification of lncRNAs involved in mouse myogenesis

To identify lncRNAs in the mouse skeletal muscle system, we used Trapnell et al.’s C2C12 myoblast and early myotube (3 days after differentiation) deep RNA sequencing (RNA-Seq) data [35]. The reads from the dataset were aligned and mapped to the mouse genome (version mm10). A total of 55,874 transcripts were identified. Protein-coding genes were excluded from this analysis. Transcripts of > 200 bp with no coding potential were selected as lncRNAs. The filtered lncRNAs were annotated by using a mouse genome annotation file. We selected lncRNAs that were temporally regulated during myoblast differentiation, as these lncRNAs may have a role or assist in myogenic differentiation. Significant lncRNAs were selected based on Log2 fold change 1 and False Discovery Rate (FDR) < =0.05, identifying 2059 differentially expressed lncRNAs in the dataset. Among the identified lncRNAs, many have been previously shown to be expressed in C2C12 cells and involved in muscle development and differentiation. We detected expression of known lncRNAs, such as *NEAT1*, *H19*, *MALAT1*, *Linc-MD1*, *MYH*, *MUNC*, *Lnc-31HG*, *LncMyoD*, *SRA1*, and *RPL12P8*, in the myotube stage, corroborating earlier studies [22, 36–38]. *Linc-MD1*, *LncMyoD*, *Malat*, and *SRA1* are involved in myoblast differentiation, whereas *Lnc-31HG* and *RPL12P8* play a significant role in myoblast proliferation. In addition to these known lncRNAs, we identified 57 conserved lncRNAs in this dataset (Table 1 and Additional file 1: Table S1). Annotation of some of these lncRNAs were found in FANTOM database [5] and these includes enhancers and promoter lncRNAs. The logCPM values derived from RNA-Seq data by Trapnell et al. were compared with the gene expression data from Liu et al. [39] and found to be highly correlated (correlation coefficient = 0.67 and *p*-value < 2.2e-16), indicating a consensus between these studies.

**Table 1:** The table shows the number of lncRNAs conserved between humans and mice. Among the 57 RNAs, 15 are lncRNAs, and the remainder are e-lncRNAs and p-lncRNAs annotated by FANTOM database. The function column shows the gene ontology of the genes associated with lncRNAs in the TAD. The functional annotation reveals that all of the identified lncRNAs are involved in developmental processes. However, some of the lncRNAs are involved in muscle development, and a few are involved in chromatin organization

Type of RNA	Number	Function		
		Chromosomal/Chromatin Organization	Developmental process	Muscle development
e-lncRNA, p-lncRNA	42	17	29	7
lncRNA	15	5	15	1

### Expression pattern of lncRNAs and nearby genes

**Fig. 1:**
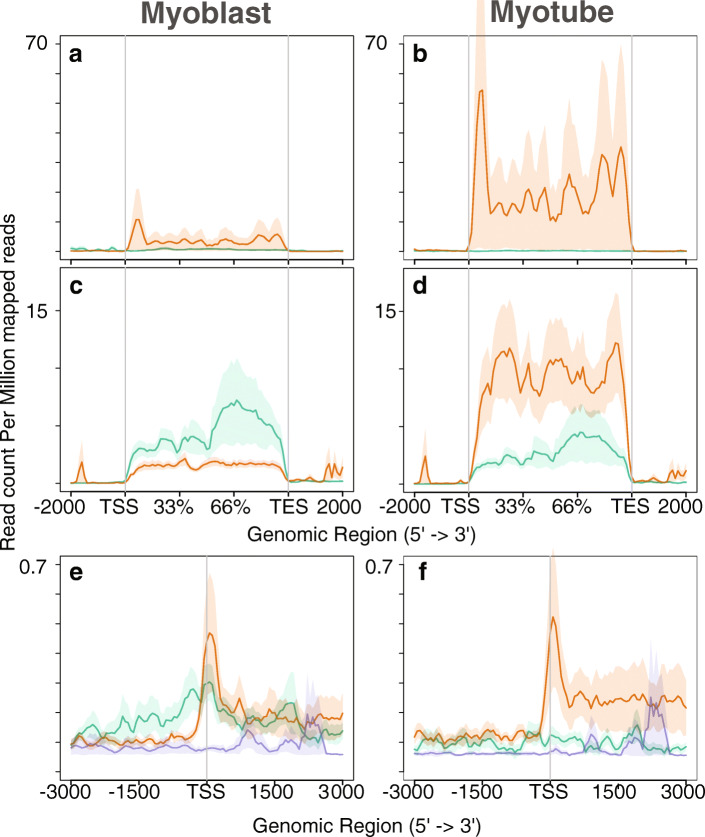
Expression pattern of lncRNAs and nearby genes correlated with PolII. **a** and **b** Expression of lncRNA. **c** and **d** Expression of nearby genes. **e** and **f** Distribution of PolII (Asp. et al.) along the identified lncRNAs from Trapnell et al. data. Green and orange plots in Fig. 1 indicate the read density of myoblast-specific and myotube-specific lncRNAs in **a** and **b** respectively. Green and orange lines indicate myoblast-specific and myotube-specific lncRNAs in **a** and **b**; nearby genes in **c** and **d**. The indigo plot (**e** and **f**) shows the non-expressing genes. Total number of 500 nearby genes taken into consideration

We observed some lncRNAs are highly expressed in the myoblast stage and decrease expression in the myotube stage; and some highly expressed in the myotube and have low expression in the myoblast stage, which is termed as myoblast-specific and myotube-specific lncRNAs, respectively. Read densities of the lncRNAs in myoblast and myotubes revealed that myotube-specific lncRNAs begin to be expressed at the myoblast stage and that levels increase during the myotube stage. We investigated nearby genes to determine the possible targets of the lncRNAs. Previous studies considered genes within 10 kb as candidate targets [40], and we observed a similar pattern with nearby genes (within the 10 kb region). Comparison between the level of myoblast-specific genes during the myoblast stage and myotube-specific genes in the myotube stage showed that the latter are expressed at a higher level than the former. Moreover, expression of myoblast-specific genes and lncRNAs decreases at the myotube stage (Fig. 1). In human dataset also, we observed a similar behaviour, highly expressed lncRNAs and genes in myoblast stage decreases at myotube stage. Genes which were highly expressed in myotube, started their expression in early stage and increases in later stage (Additional file 2: Figure S1). In mouse, we did not observe a higher expression of myoblast-specific genes and lncRNAs in myoblasts because we considered the later stage of myoblasts. At this stage, myoblast-specific gene expression is destined for silencing, and myotube-specific genes are triggered for expression.

We compared the observed expression pattern with the distribution of RNA polymerase II (PolII) (Fig. 1 e and f) using the PolII binding profile obtained from Asp et al.’s study [41]. As PolII is involved in lncRNAs expression, we investigated the distribution of PolII at the TSS of selected lncRNAs and nearby genes and observed a similar pattern of distribution. Specifically, we found considerable enrichment of PolII on myotube-specific genes in myoblasts (Fig. 1e), suggesting that these gene regions had already been converted to active chromatin.

### Comparative analysis and identification of conserved lncRNAs in mouse and human

**Fig. 2:**
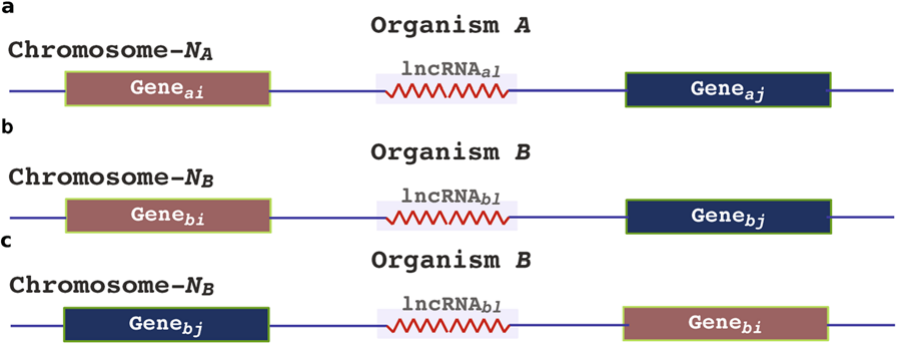
The schematic representation of the criteria for determining the conserved lncRNAs in mouse and human datasets. **a** shows the identified lncRNA in mouse. **b** The corresponding lncRNA in humans. In humans, there can be two scenarios. In b), the synteny remains the same; however, in **c**, the gene arrangements were changed

In mammals, muscle development occurs through distinct myogenic waves and is evolutionarily conserved. Moreover, transcription factors responsible for the commitment of mesodermal cells to a muscle lineage and the initiation and maintenance of the terminal differentiation programme are highly conserved in mammals [42]. To identify lncRNAs conserved between mice and humans,

we matched the lncRNAs identified from mice with those in humans using Zeng et al.’s RNA-Seq data [43], which are comprehensive single-cell and single-nucleus RNA sequencing data generated to study gene expression profiles in undifferentiated myoblasts and myotubes (72 h after induction of differentiation) in Hu5/KD3 (KD3) cells. Pairwise sequence comparison of the lncRNAs from humans and mice revealed very weak conservation (Additional file 3: Figure S2). Therefore, to find conserved lncRNAs, we first selected the neighbouring genes (upstream and downstream) of the mouse lncRNAs as a reference point (Fig. 2). These genes were delineated in the human genome, after which we investigated whether any lncRNAs are located near these reference genes in the human dataset. Thus, we identified common lncRNAs in mice and humans based on the reference genes. To re-verify the sequence conserveness, we have annotated the lncRNA conserved sequence alignment information among 100 vertebrates species by using the MULTIZ alignment program provided in the RNA-Central database(v14, [44]). While six of the identified lncRNAs (RP11-887P2.5, RP11-366 L20.2, LINCMD1, CARMN, AC007383.3, MALAT1) were highly conserved across species (mean phastcon score ranges from 0.80 to 0.99), most of them showed moderate to poor conservation (Additional file 4: Table S2). Further, the consensus structure of the lncRNAs was built by using CRS database (Additional file 4: Table S2 and Additional file 5: Figure S3). The database holds the information of vertebrate genomes for conserved RNA structures and consensus structure was built based on the CMfinder program using the expectation-maximization algorithm using covariance models [34]. Some of these lncRNAs showed consensus secondary structure, which implies that they may have some common role to play in myogenesis.

While analysing these common lncRNAs, we found some known common lncRNAs, such as *NEAT1, XIST,* and *H19*. We also noticed that the synteny around the lncRNAs is conserved between mice and humans. Overall, we identified 57 conserved lncRNAs (Table 1). Of these 57 lncRNAs, there were only 13 with nearby genes within the 10-kb window, and the remaining 44 were in gene desert regions. The conserved location and moderate structural conservation may indicate a common role of these lncRNAs in both the organism.

Because a large number of RNAs are located in gene desert regions, we examined whether they are in enhancers. To determine the enhancer’s property, we utilized the H3K27Ac ChIP-seq profiles from the Bernstein lab of the Broad Institute’s Human Genome Project [2]. We assessed the level of H3K27Ac in skeletal muscle myoblasts in humans, as well as H3K27Ac levels in other cell lines, such as GM12878, H1-hESC, and K562 cells. This analysis revealed that many of the RNAs are located in enhancer regions. We further investigated whether the lncRNAs overlapped with the regulatory elements within 5 kb region by integrating CTCF binding sites, promoters, proximal enhancers, distal enhancers from ENCODE for both human and mouse genome. Significant amount of overlapped was found for enhancers regions (proximal and distal enhancers) compared to promoters and CTCF binding sites (Additional file 6: Figure S4). The complete lists of genomic position of lncRNAs with each of the regulatory elements for human and mouse datasets provided as supporting information (Additional file 7).

However, we also found that a few RNAs are located in gene desert regions that do not carry the enhancer-specific marker H3K27Ac. One reason for the lack of H3K27Ac marks may be that we have yet to detect the deposition of H3K27Ac marks in C2C12 or KD3 cells or other cell lines; another reason may be that these sites are not typical/canonical enhancers. These sites are distal regions in one-dimensional space but may be closer in three-dimensional space. Moreover, we detected multiple possible lncRNAs in humans for only a few lncRNAs in mice, though we selected only one among the multiple hits based on the distance and log-fold change as well as the expression level. The sequence of these common lncRNAs is not conserved; however, as they are expressed in both mice and humans, they likely have an important role in the structural conformation of the genome during differentiation.

### Correlation of the lncRNA expression pattern with epigenetic factors

**Fig. 3:**
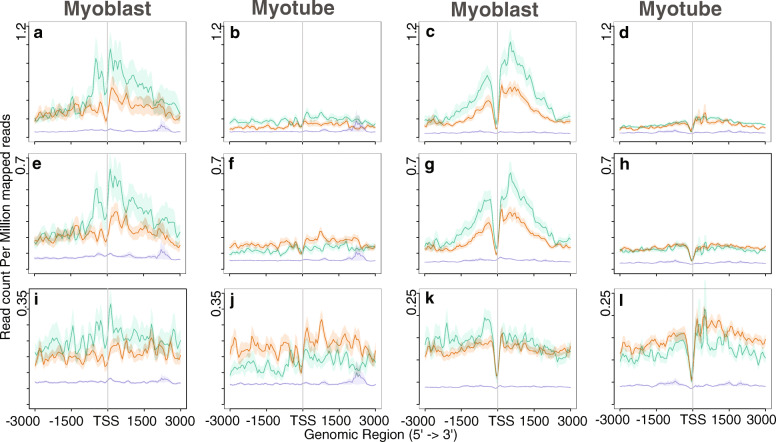
Distribution of Acetylation of histones across lncRNAs and nearby genes. **a** and **b** Distribution of H3K9Ac in lncRNAs. **c** and **d** Distribution of H3K9Ac in nearby genes. **e** and **f** Distribution of H4K12Ac in lncRNAs. **g** and **h** Distribution of H4K12Ac in nearby genes. **i** and **j** Distribution of H3K18Ac in lncRNAs. **k** and **l** Distribution of H3K18Ac in nearby genes. Green lines indicate myoblast-specific lncRNAs and nearby genes. Orange lines represent myotube-specific lncRNAs and nearby genes. Indigo lines indicate gene having no expression. Total number of 500 nearby genes taken into consideration

**Fig. 4:**
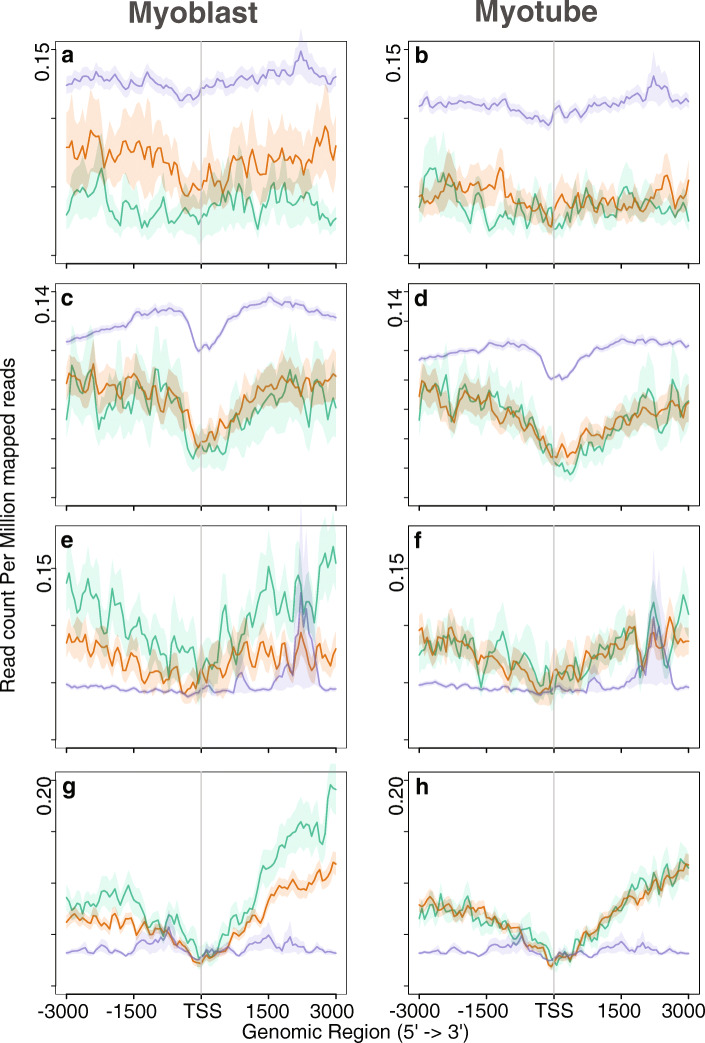
Distribution of tri-methylation in H3K27 and H3K36 across lncRNAs and nearby genes. **a** and **b** Distribution of H3K27me3 in lncRNAs.**c** and **d** Distribution of H3K27me3 in nearby genes. **e** and **f** Distribution of H3K36me3 in lncRNAs. **g** and **h** Distribution of H3K36me3 in nearby genes. Green lines indicate myoblast-specific lncRNAs and nearby genes. Orange lines represent myotube-specific lncRNAs and nearby genes. Indigo lines indicate gene having no expression. Total number of 500 nearby genes taken into consideration

**Fig. 5:**
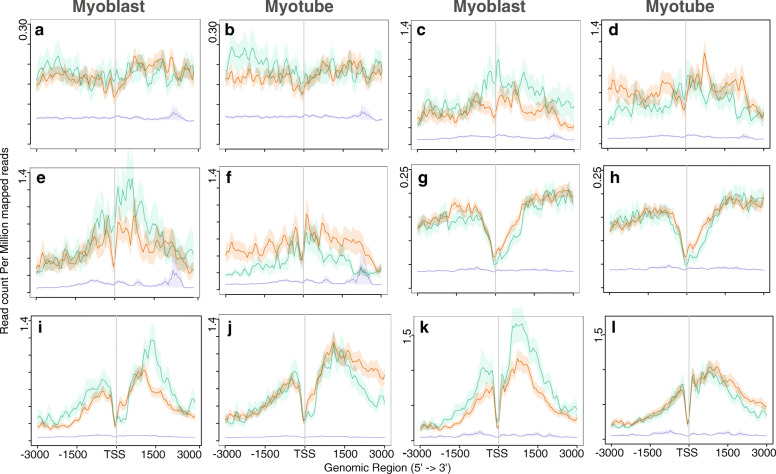
Distribution of methylation on H3K4 across lncRNA and nearby genes. **a** and **b** Distribution of H3K4me1 in lncRNAs. **c** and **d** Distribution of H3K4me1 in nearby genes. **e** and **f** Distribution of H3K4me2 in lncRNAs. **g** and **h** Distribution of H3K4me2 in nearby genes. **i** and **j** Distribution of H3K4me3 in lncRNAs. **k** and **l** Distribution of H3K4me3 in nearby genes. Green lines indicate myoblast-specific lncRNAs and nearby genes. Orange lines represent myotube-specific lncRNAs and nearby genes. Indigo lines indicate gene having no expression. Total number of 500 nearby genes taken into consideration

Because the regulation of gene expression during lineage commitment and differentiation is controlled by dynamic changes in chromatin, we investigated histone modifications that play an essential role in chromatin architecture. To this end, we examined the histone modification profiles obtained by Asp et al. [41] along with lncRNAs and nearby genes (Figs. 3, 4 and 5). Asp et al. performed genome-wide mapping of histone modifications to investigate changes in chromatin during the differentiation of myoblasts into myotubes. The distribution pattern of modified histones for the lncRNAs was found to roughly follow the same pattern as that for nearby protein-coding genes. For example, the distribution pattern of H3K9Ac and H4K12Ac in myoblasts peaked around the TSS for both nearby genes and lncRNAs (Fig. 3 a-h). In myotubes, these levels decreased around the TSS. However, a similar pattern was not observed for the H3K18Ac mark (Fig. 3 i-l): unlike H3K9Ac and H4K12Ac, we did not observe sharp peaks around the TSS for H3K18Ac, and the level did not decrease in myotubes. H3K18Ac deposition on myoblast-specific genes remains the same but increases slightly on myotube-specific genes. Asp et al. observed that H3K18Ac levels decrease on constitutively expressed genes with lower expression in myotubes. However, our observations suggest that the genes selected in this study are not constitutively expressed. As observed by Asp et al., we also found that the distribution of H3K18Ac was not restricted to regions surrounding the TSS (Fig. 3 i-l).

While investigating the repressive marker H3K27me3, we observed a low level on genes expressed during myogenesis (Fig. 4 a-d). In contrast, non-expressed genes exhibited a higher level of H3K27me3 deposition. Supporting this observation, we did not detect accumulation of PolII at these non-expressing genes (Fig. 1 e and f).

Genome wide mapping of histone deacetylase (HDAC) and Histone acetyltransferases (HATs) in human genome indicated that H3K4 methylation primes chromatin to facilitate histone acetylation and H3K36me2/3 facilitates deacetylation slows elongation [45]. We observed low deposition of H3K36me3 at the TSS of both lncRNAs and nearby genes (Fig. 4 e-h), suggesting that these genes were expressed. Although the level of H3K36me3 remained the same for myotube-specific genes, the level decreased for myoblast-specific genes. As expected, the level of activation marker H3K4 methylation was high at the promoters and gene bodies of active genes (Fig. 5). We observed similar distribution pattern of histones marks in human dataset (Additional file 8: Figure S5, Additional file 9: Figure S6). We overlapped the modified histone marks within 5 kb region of the lncRNA for both mouse and human genome. We observed that the distribution pattern of the modified histone marks in mouse and human are conserved (Additional file 10: Figure S7 and Additional file 11).

### Quantitation of lncRNAs in myoblasts and myotubules

**Fig. 6:**
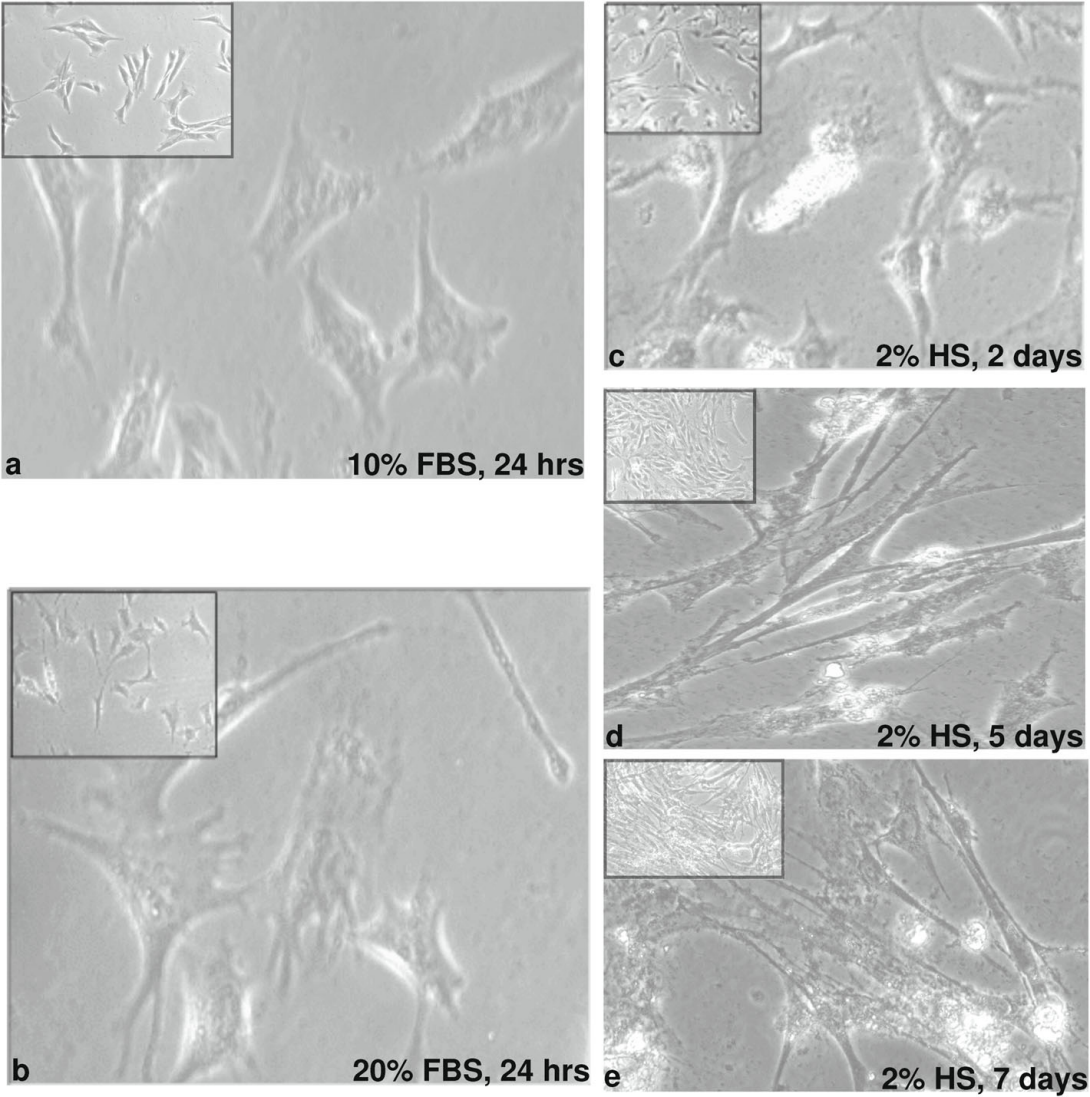
Morphological changes in C2C12 cells during myogenic differentiation. **a** Light microscopic images of myoblasts (10% FBS) 20X magnification (10x magnification in inset). **b** Light microscopic images of exponentially proliferating myoblasts (20% FBS). **c** differentiating myotubes after serum deprivation (2% Horse Serum) for 2 days. **d** differentiating myotubes after serum deprivation (2% Horse Serum) for 5 days. **e** differentiating myotubes after serum deprivation (2% Horse Serum) for 7 days of media change and induction of differentiation

We cultured C2C12 skeletal muscle myoblast cells to monitor the differentiation. (Fig. 6a). The cells actively divided and displayed a very clear myoblast morphology (Fig. 6b). The changes in their morphology were monitored at 2 days (Fig. 6c), 5 days (Fig. 6d) and 7 days (Fig. 6e). The cell morphology towards that of a myotube over time, showing a myotube-like morphology on day 7. This result clearly indicates in vitro differentiation of myoblasts into myotubules. Further to evaluate the differentiation of the C2C12 cells, we quantified the expression of well-known genes involved in the myogenesis process. We quantified the expression of Myf5 and MyoG gene in mouse C2C12 cells. The expression patterns of these genes signify the differentiation of C2C12 cells (Additional file 12: Figure S8).

**Fig. 7:**
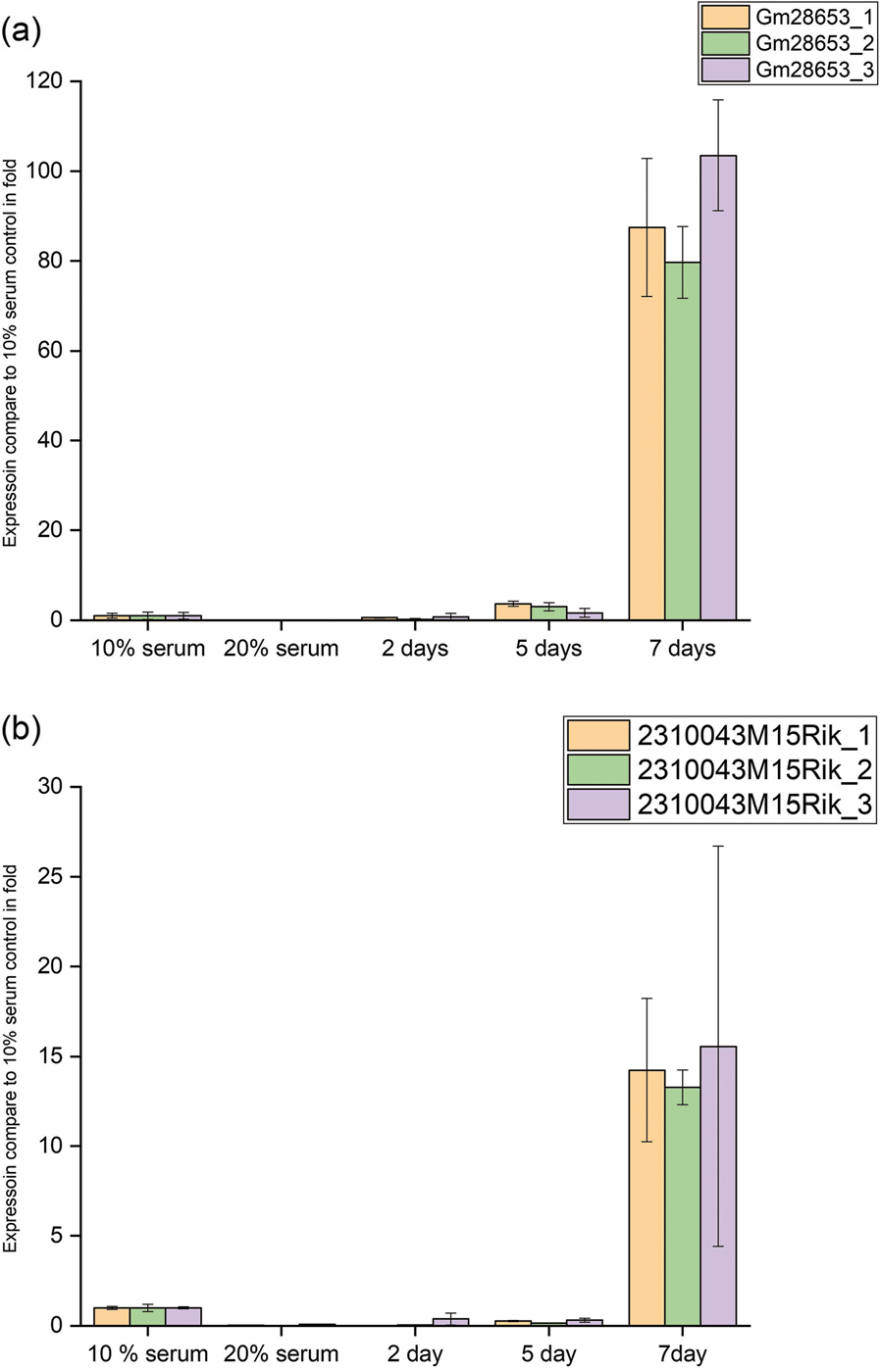
Expression of muscle associated long non-coding RNA (lncRNA) determined by quantitative RT-PCR at different stage of differentiation. **a** Expression levels of lncRNA Gm28653 by using three different primers 53–1, 53–2 and 53–3 **b** Expression level lncRNA 2310043M15Rik by using three different primers Rik-1, Rik-2 and Rik-3 during myoblasts culture with 10% FBS, exponentially proliferating myoblasts in 20% FBS as well as differentiating myotubes at 2, 5 and 7 days after serum starvation

To quantify the computationally identified lncRNA expression in mouse C2C12 cells, we measured two lncRNAs: *Gm28653* and *2310043M15Rik*. C2C12 cells were collected at different stages of differentiation, total RNA was isolated, and expression of both lncRNAs was quantitated by reverse transcriptase PCR (RT-PCR); Gapdh and β-actin were used as loading controls to which all samples were normalized. Three primers were used for each: *Gm28653,* 53–1, 53–2 and 53–3; *2310043M15Rik,* Rik-1, Rik-2 and Rik-3. All primers showed unique products, as determined by a single peak of the melting curve (Additional file 13:Figure S9), and all the samples were normalized relative to the 10% BSA control. Expression of lncRNA *Gm28653* was down-regulated in myoblasts growing in 20% FBS, but its level gradually increased over time, with maximum expression on day 7 (Fig. 7a). For *2310043M15Rik,* it was also down-regulated in 20% FBS, suggesting lower expression in myoblasts. However, its expression increased with the duration of low-serum treatment, with the highest level also on day 7. These experiments reflect the association of these lncRNAs with the myogenic differentiation of C2C12 cells (Fig. 7b).

### Association of lncRNAs with genes in 3-dimensional space

Few earlier methods have been able to assign the function of lncRNAs based on the activity of nearby protein-coding genes [31] or co-expressed neighbouring coding genes [32, 33], which may be helpful if the lncRNA and protein-coding gene are close to each other. In this study, many of the identified lncRNAs were found to be distally located in gene desert areas. Therefore, previous approaches may not be applicable for assigning function based on nearby genes. Nonetheless, if we consider the three-dimensional chromatin architecture, it may be possible to identify domains where genes and selected lncRNAs are close to each other in three-dimensional space. Dixon et al. [46] generated Hi-C experimental data related to the chromatin structure in mammalian cells, dividing the genome into smaller blocks, modules or domains based on the distance or positional association of the genomic fragments. These domains are termed topologically associated domains (TADs). From this dataset, TADs were identified and overlapped with the location of our lncRNAs. Dixon et al. studied chromatin structures in pluripotent cells, such as mouse embryonic stem cells (mESCs) and human embryonic stem cells (hESCs), and differentiated human IMR90 fibroblasts; they observed that the overall domain structure between cell types is mostly unchanged in both pluripotent cells and their differentiated progeny. Therefore, in our analysis, we first identified TADs from mESCs and overlapped the location of the lncRNAs identified in mouse C2C12 cells, which gave us the associated genes with these lncRNAs in three-dimensional space. Gene ontology study revealed that lncRNAs and associated genes in the TADs are involved in skeletal muscle development process, muscle cell proliferation, muscle cell differentiation, chromosome organizations, histone modifications, developmental process, cellular component organizations. Some of the lncRNAs and associated genes are involved in immune response, metabolic process, cell signaling, multicellular organism development (Additional file 14).

### Functional assessment of identified lncRNAs

Ling-Ling Zheng et al. used co-expression strategy to predict the putative function of lncRNAs [47]. Xiaoyue Li et al. combined Gene ontology and an approach of identification of nearby genes (100 kb) that are potentially regulated by lncRNAs [48]. We have integrated three dimensional architecture of the genome (Hi-C), Gene enrichment analysis and nearby genes co-expression strategy to identify the potential functions of the lncRNAs.

**Fig. 8:**
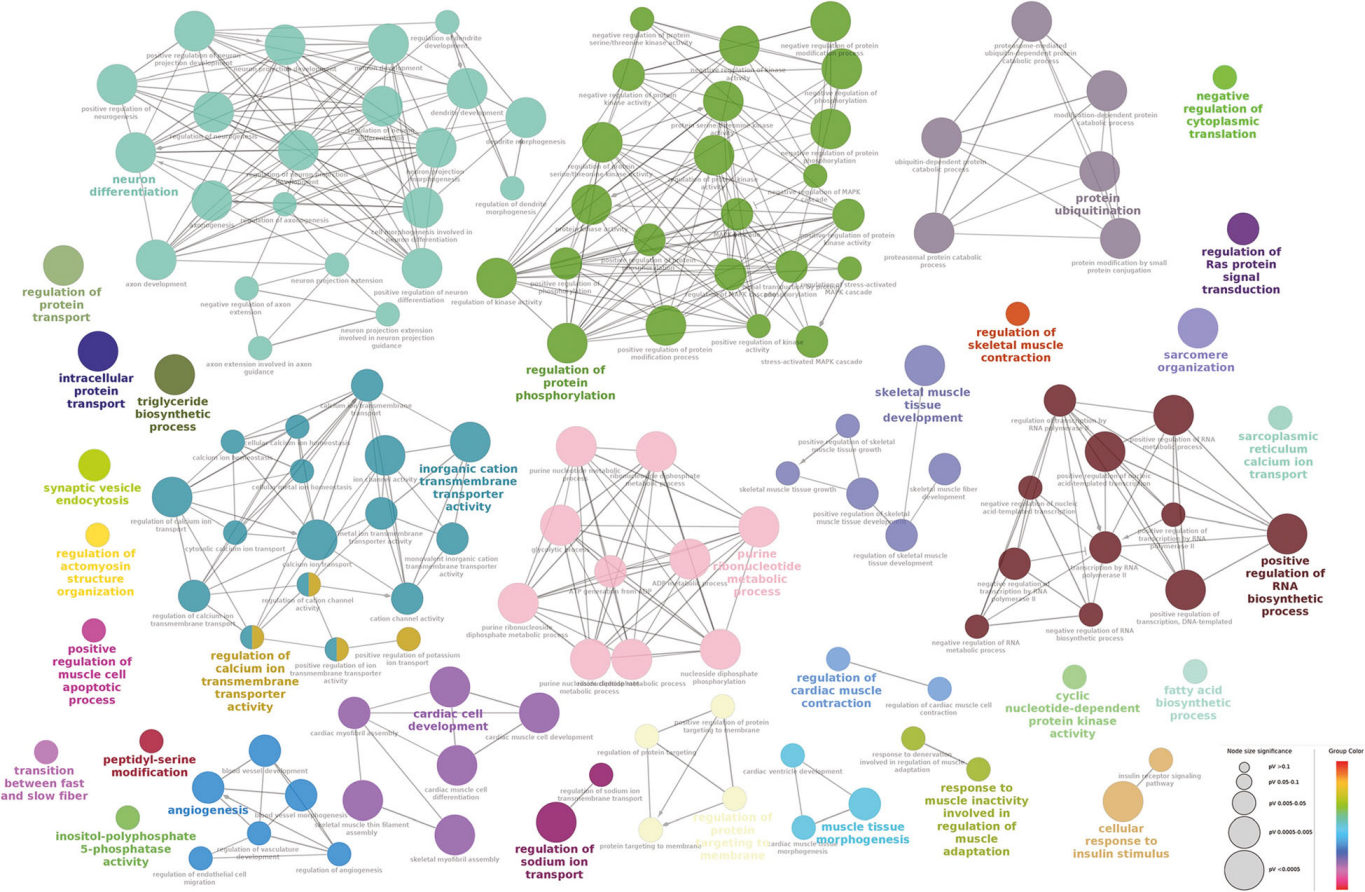
Gene enrichment analysis of differentially expressed genes in myotubes. ClueGO analysis of enriched Biological Process (BP) Gene Ontology network of genes expressed in myotube. The nodes represent the BP and the edges denote their connections. Size of the node denotes the enrichment significance and color denotes its class. Nodes with mixed colors denote multiple classes. The most significant term in the network is highlighted in bold

**Fig. 9:**
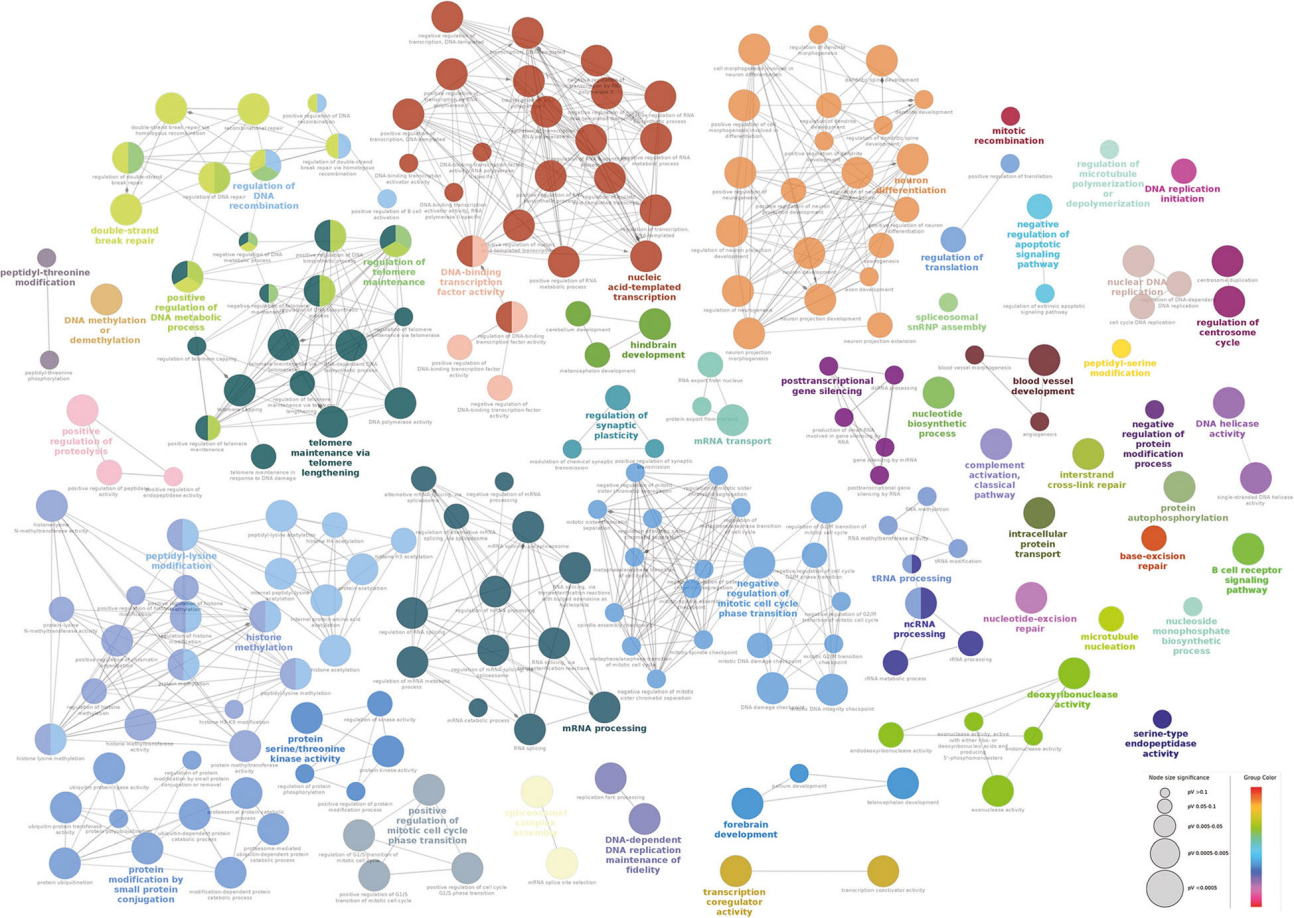
Gene enrichment analysis of differentially expressed genes in myoblast. ClueGO analysis of enriched Biological Process (BP) Gene Ontology network of genes expressed in myoblast stage. The nodes represent the BP and the edges denote their connections. Size of the node denotes the enrichment significance and color denotes its class. Nodes with mixed colors denote multiple classes. The most significant term in the network is highlighted in bold

Analysis of the differentially expressed nearby protein-coding genes in myotube stage revealed that pathways such as regulation of skeletal muscle adaptation, regulation of myotube differentiation, skeletal muscle organ development, etc. were found to be prominent (Fig. 8). However, in the myoblast stage, we observed transcription and cell cycle-related pathways (Fig. 9). The complete list of associated genes involved in pathways along with *p*-value is provided as supporting information (Additional file 15). However, TADs were utilized to investigate the expression and function of genes associated with selected lncRNAs, revealing genes in the same TADs to be co-expressed, as previously observed by Soler-Oliva et al. [49]. Genes in TADs containing lncRNAs were mostly enriched in ontologies related to the cell cycle, glucose metabolism, lipid metabolism, cytoskeleton, actin filament, development and differentiation, and transcription.

Genes in TADs upregulated in myotubes were enriched in skeletal muscle development and function, and those upregulated in myoblasts are mainly involved in cell cycle progression and mitosis, which is a characteristic of proliferating myoblasts. Some well-studied lncRNAs involved in genome organization were found to be very highly expressed. For example, *H19* is expressed in a markedly higher order. It has been reported that *H19* is highly expressed during foetal development and is involved in myocyte glucose uptake, embryonic development and muscle regeneration using chromatin modifiers [37, 50, 51]. With our approach, we discovered a similar function. Indeed, the genes in the TAD containing *H19* are involved in histone modification, glycogen biosynthesis, mitotic nuclear division, muscle cell differentiation, cellular protein metabolism, and muscle contraction, and sliding. Similarly, the genes in the TADs of *Neat1* and *Malat1* were found to be associated with the developmental process and chromosome organization. Earlier studies have demonstrated that *Neat1* and *Malat1* are essential for the structural integrity of nuclear paraspeckles [52]. In mammalian cell nuclei, the ribonucleoprotein bodies known as paraspeckles play an essential role in regulating the expression of certain genes in differentiated cells through nuclear retention of RNA [53]. These findings indicate that our approach can act as a preliminary method to identify the function of lncRNAs.

In addition to *Neat1*, *Malat1* and *H19*, we discovered some lncRNAs involved in chromatin organization, for example, *Bach2os, Srrm4os, Gm21747, Gm17518,* and *Gm43672.* The TAD containing *Gm14635* was found to be populated with H2A histone family genes involved in biological processes and negative regulation of histone H3-K36 methylation. One TAD containing lncRNA *Hist2h2bb* was identified as containing replication-dependent histone genes along with immune and transcription-related genes. These lncRNAs may also be involved in chromatin reorganization.

Two of the lncRNAs detected, *Gm29237 and Gm20342,* participate in the cell cycle and metabolic processes in addition to chromatin remodelling (Additional file 1: Table S1). Other lncRNAs were previously demonstrated to be involved in dephosphorylation, intracellular signal transduction, cation transport, and the import of protein into the nucleus mechanism. However, using our approach, we could not assign functions to some lncRNAs, as we were unable to find any associated genes in the TADs (Additional file 1: Table S1).

## Discussion

As evidence of the involvement of lncRNAs in the regulation of gene expression in a number of biological processes grows, it is essential to investigate the participation of lncRNAs in different mechanisms. In the current study, we examined expression patterns of lncRNAs and identified conserved lncRNAs involved in myogenesis. We integrated RNA-Seq, ChIP-Seq, regulatory elements, and Hi-C datasets for the detection of differentially expressed lncRNAs and their association with different histone marks, and identified conserved lncRNAs in mouse and human. We designed a criteria to determine conserved lncRNAs across species, and also shed light on the role of these lncRNAs based on the three-dimensional architecture of the genome. This approach revealed that the conserved lncRNAs may play an important role during myogenesis. We first determined lncRNAs differentially expressed in the mouse and human genomes. Regarding expression patterns, we observed a substantial increase in the expression of myotube-specific lncRNAs after differentiation and a decrease in myoblast-specific lncRNAs in differentiated myotubes. The same pattern was detected for nearby genes. Intriguingly, we observed significant up-regulation of myotube-specific lncRNAs and nearby genes in myoblasts. We investigated PolII deposition along the TSSs of nearby genes and lncRNAs, and our findings corroborated the observed expression patterns for lncRNAs. Accordingly, these lncRNAs likely had already adopted features of active chromatin before maximal expression in myotubes. This observation was confirmed by the distribution of the trimethylation marker H3K36 in the gene bodies of lncRNAs and nearby protein-coding genes, which signifies the active transcription of genes by RNA PolII. We observed a notable decrease in the level of PolII along myoblast-specific lncRNAs in myotubes and a decrease in histone acetylation during myogenic differentiation. As illustrated in Fig. 3, the levels of H3K9Ac and H4K12Ac decreased drastically in myotubes. Earlier studies have reported a similar pattern [41] and confirmed that the N-terminal tail of this histone is cleaved (at residues 22–23) during ES differentiation [41, 54]. This cleavage of histones upon differentiation may explain why we observed a decreased level of these modifications in myotubes. Regardless, we did not detect significant global changes in the trimethylation of H3K4, H3K36, and H3K27 during differentiation (Figs. 4 and 5). Overall, our analysis suggested that the observed expression pattern of lncRNAs correlated with epigenetic marks.

Recent studies have reported that more than 90% of the genome giving rise to RNA [3] is non-functional [2, 55], originating from transcriptional noise or the artefacts of sensitive detection methods [56]. To validate the computationally identified lncRNAs in our study, we selected two for experimental verification in C2C12 cells based on their level of expression. Among the lncRNAs detected, known lncRNAs, such as *XIST* and *MALAT*, were highly expressed. In contrast, the lncRNAs detected in our study were found to be expressed at a very low level. To determine whether these lncRNAs indeed participate in myogenesis, we cultured C2C12 cells and quantitated expression of Gm28653 and 2310043M15Rik lncRNAs by RT-PCR, finding that the levels were similar to those observed in the computational analysis. Moreover, some of the identified lncRNAs were found to be conserved between humans and mice (Additional file 1: Table S1), suggesting that they are non-random muscle-specific biologically functional lncRNAs.

Because the identified lncRNAs are conserved, we determined their possible function. The function of lncRNAs has been inferred by exploring relationships between lncRNAs and nearby protein-coding genes [31], and functions have been predicted by identifying coding genes co-expressed with lncRNAs [32, 33]. In this study, we first investigated the gene ontology of nearby genes because lncRNAs are known to exhibit enhancer-like transcription-dependent activation or repression of neighbouring protein-coding genes [6, 57]. The overall gene ontology of the nearby genes showed enrichment in cellular processes, metabolic processes, biological regulation and developmental processes in both human and mouse datasets.

In addition to assign a function to the lncRNAs, we investigated lncRNAs in light of chromatin three-dimensional architecture data from Dixon et al. [46]. The ontologies of genes sharing the same topological domain with lncRNAs can help determine the role of the lncRNAs. One of the mechanisms by which lncRNAs control gene expression is the scaffold transcript, which provides binding sites for several RNA-binding proteins that can recruit chromatin-modifying enzymes [58, 59]. For example, *HOTAIR* can recruit Polycomb Repressive Complex 2 to its 5′ end, followed by the generation of the H3K27me3 silencing mark, whereas its 3′ terminus can interact with the LSD1/CoREST/REST complex [59]. In the present study, we detected previously reported lncRNAs that form a scaffold for chromatin architectural changes, such as *NEAT1*, *XIST*, *Malat1,* and *H19* (Table 1). The ontologies of the genes co-existing in TADs are involved in chromatin and chromosome organization, covalent chromatin modification, regulation of chromatin organization, regulation of histone modification, and differentiation and development. We discovered 22 lncRNAs that colocalized with the chromatin-modifying genes in the same TAD, suggesting that these lncRNAs may have a similar mechanism of gene regulation as *NEAT*, *XIST*, and *MALAT1*, among others (Additional file 1: Table S1). The occurrence of lncRNAs and chromatin-modifying genes in the same TAD may suggest that the protein product of these genes is recruited by the lncRNA, which is nearby. Most of the lncRNAs we identified as being common between mice and humans are located in enhancers. It has been reported that enhancer RNAs [57, 60] are transcribed from enhancers and control gene expression by affecting looping between enhancers and promoters [61, 62]. Therefore, these lncRNAs in enhancers may adopt mechanisms of nucleosome positioning, chromosome looping, guide or decoy lncRNAs. Although whether the mechanisms by which lncRNAs control chromatin structure are conserved across species has yet to be determined, it is clear from our study that lncRNAs are conserved between different species, despite very low sequence similarity. This conservation may indicate that the process of chromatin structure control by the identified lncRNAs is mechanistically conserved among species. Such changes in chromatin organization directly affect transcription factor binding and RNA polymerase activity. Nonetheless, it is difficult to suggest which mechanism the remaining lncRNAs (Additional file 1: Table S1) adopt. Additionally, we discovered that some lncRNAs are engaged in cell cycle processes and metabolism. To confirm that these lncRNAs are specific to myogenesis, we cross-checked their expression in NONCODE [63] and GTEXPortal [64] and found that 21 of 57 lncRNAs are also expressed in the heart, hippocampus, liver, lung, spleen, and thymus. The remaining lncRNAs might be more specific for muscle.

## Conclusions

In summary, by integrative data analysis approach, we identified 57 differentially conserved lncRNAs in humans and mice. Studies are required to investigate the reason for the conservation of lncRNAs in humans and mice, even though their sequences are dissimilar. The lack of conservation of the lncRNA sequences may indicate that the mechanism of the lncRNAs recruiting other proteins using the motif-based binding is unlikely. However, since these lncRNAs have moderate structural conservation, chromatin structural changes may be introduced by these lncRNAs, which may regulate chromatin accessibility by the transcriptional machinery. Our analysis provides insight on the conservation of lncRNAs between human and mice and their functional annotation in myogenesis.

## Methods

Myogenic transcriptomic data were analysed to identify differentially expressed lncRNAs. The filtered and processed reads were aligned to the reference genome. We then estimated the transcript abundances of the alignments using RNA-Seq Expectation-Maximization Method and selected differentially expressed genes with at least a 1-fold change and (False Discovery Rate correction <=0.05) for downstream analysis. Expression was correlated with the histone modification study performed using Chip-seq datasets for H3k4me2, H4k20me1, H3k4me3, H3k4me1, H3k27me3, H3k36me3, H3k9ac, H3k79me2, H3k9me3, and H3k27ac for myoblasts and myotubes. Additionally, we performed real-time quantitative PCR to verify expression of muscle-associated lncRNAs at different stages of differentiation. Finally, we identified lncRNAs conserved between humans and mice and assessed their functional roles by overlapping the lncRNAs in TADs and investigating the ontologies of associated genes.

### File processing and quality control of the dataset

Raw SRA files were converted to FASTQ files using SRA Toolkit. Low-quality reads and adapter sequences were trimmed, and other sequencing errors (polyX detection, overlapping) were removed using the AfterQc program(v0.9.7, [65]). The program identified low quality reads if it meet at least one of following criteria: 1) too high or too low of mean base content percentages (i.e. higher than 40%, or lower than 15%); 2) too significant change of mean base content percentages (i.e., ±10% change comparing to neighbour cycle); 3) too high or too low of mean GC percentages (i.e. higher than 70%, or lower than 30%); 4) too low of mean quality (i.e. less than Q20).

### Alignment of the reads

The processed reads were aligned by the RNA-Seq aligner STAR(v2.5) [66] with the Ensemble Human reference annotation (GRCh38) and Ensemble Mouse reference annotation (MM10), respectively. We used the parameter ‘–outFilterMismatchNmax 10 –outFilterMismatchNoverReadLmax 0.07 – outFilterMultimapNmax 10’ to accurately align the reads and identify lncRNAs [43]. The STAR aligner is suitable for aligning longer reads with high mapping accuracy and is designed to align non-contiguous sequences directly to the reference genome, which contributes to transcriptome studies by providing more complete RNA connectivity information.

We used two approaches, namely, “stringent” and “relaxed”, to identify lncRNAs from the datasets. In the stringent approach, we used the parameter “–outFilterMismatchNmax 10 –outFilterMismatchNoverReadLmax 0.07 – outFilterMultimapNmax 10” during the alignment, and short reads were removed. The “relaxed” approach aligns all reads irrespective of their length. In the “relaxed” approach, we used the argument “--outFilterScoreMinOverLread 0 --outFilterMatchNminOverLread 0 --outFilterMatchNmin 0 --outFilterMismatchNmax 10 --outFilterMismatchNoverReadLmax 0.7 --outFilterMultimapNmax 10” .

We detected more lncRNAs (613) in the “relaxed” approach than in the “stringent” (204) approach. A total of 150 lncRNAs overlapped between the two methods, and these lncRNAs were found to be statistically significant (FDR < =0.05).

### Quantifying transcript abundances from datasets

We used RSEM (RNA-Seq by Expectation Maximization) (v1.2.31) to quantify gene and isoform abundances from the paired-end RNA-Seq dataset. RSEM is a software package for quantifying gene and isoform abundances from single-end or paired-end RNA-Seq datasets that computes maximum-likelihood abundance estimates using the expectation-maximization (EM) algorithm as its statistical model. The directed graphical model can represent the statistical model used by RSEM. After convergence, RSEM outputs ML values, as well as the expected value of the number of RNA-Seq fragments derived from each transcript, given the ML parameters. A typical run of RSEM consists of just two steps: generation of a set of reference transcript sequences and alignment to reference transcripts. The resulting alignments are used to estimate abundances and their credibility intervals [67].

The reference genome for human (version GRCh38) and mouse (version MM10) were built by using the rsem-prepare-reference script. We then used STAR to perform transcriptome-based mapping, and gene expression was calculated from STAR-generated BAM files by rsem-calculate-expression scripts.

Liu Y et al. [39] used a C2C12 cell line model to study myogenesis and regeneration, whereby the cells were allowed to differentiate from myoblast precursor cells into myotubes, followed by identification of genes that were up-regulated and down-regulated during the differentiation process. The microarray datasets were filtered based upon the presence of expressed genes in both myoblasts and myotubes. Genes that were not expressed were not considered for correlation analysis. The expression values of microarray datasets were normalized by subtracting the expression values of genes in myoblasts from those in myotubes and comparing with the log fold change values from Trapnell et al.’s data. Correlation analysis revealed a positive correlation between the genes expressed in myogenesis by microarray-based methodology and Trapnell et al.’s methodology (RNA-Seq), with a correlation value of 0.67.

### Identification of differentially expressed lncRNAs

The edgeR package(v3.30.0) [68], which depends upon count-based expression data for determining differential expression, in R was used for differential gene expression analysis. An overdispersed Poisson model was used to account for both biological and technical variability. Empirical Bayes methods are used to moderate the degree of overdispersion across transcripts, improving the reliability of inference. We removed transcripts with zero expression in the samples for both the human and mouse models. Normalization of the counts was performed by using the calcNormFactors function of edgeR, which normalizes for RNA composition by finding a set of scaling factors for the library sizes that minimize the log-fold changes between samples for most genes [68]. We used the TMM method of normalization for both datasets.

### Cell culture and myogenic differentiation

The myogenic mouse C2C12 cell line was maintained in growth medium, i.e., Dulbecco’s modified Eagle’s medium (DMEM) supplemented with 10% foetal bovine serum (FBS) and 25 mM Hepes, in a 5% CO_2_ atmosphere at 37 °C and 95% humidity. For rapid proliferation, the medium was changed to 20% FBS for 24 h, and 70–80% confluence was attained. The proliferating cells were then switched to a differentiation medium (DMEM containing 2% horse serum) that was subsequently changed every 48 h. Samples were taken on day 2, day 5 and day 7.

### RNA isolation and cDNA synthesis

For extraction of total RNA, cells were collected during growth in 10% FBS and at the exponentially growing phase under high-serum conditions (20% FBS) as well as at 2 days, 5 days and 7 days after the medium shift with differentiation medium. RNA was isolated using RNeasy Mini Kit (Qiagen, USA) according to the manufacturer’s protocol. cDNA was prepared from 1 μg of total isolated RNA using an iscript cDNA synthesis kit (BIO-RAD, USA).

### Real-time quantitative PCR

Real-time quantitative RT-PCR was carried out using SYBR green (SOLIS BIODYNE, EUROPE). Samples were run in triplicate with 7.5 ng of cDNA with custom-designed lncRNA primers and a STEP ONE PLUS™ REAL TIME PCR system (Applied Biosystems). The sequences of the 9 primers are provided (Additional file 16: Table S3).

The PCR programme consisted of a holding stage of 95 °C for 15 min, followed by 45 cycles of 15 s at 95 °C, 20 s at 60 °C and 30 s at 72 °C, with 1 h of melting curve stage. GAPDH and ß-Actin were used as internal controls. Relative expression was determined using the eq. 2^−dCT^, where dC_T_ = (C_target_ − C_control_).

### Annotation of differentially expressed transcripts

DE transcripts were selected based on log2 transformation Fold Change + − 2 and False Discovery Rate < =0.05. lncRNAs and protein-coding genes were taken into consideration based on these criteria. The lncRNA length > =200 bp was further taken into consideration. The transcripts were annotated using the Ensemble Human reference annotation (GRCh38) GTF file and Ensemble Mouse reference annotation (mm10) GTF file for the human and mouse datasets, respectively. The coding potential of DE lncRNAs was analysed using Coding Potential Assessment Tool (CPAT) [69], which applies a logistic regression model that rapidly recognizes coding and noncoding transcripts from a large pool of candidates. The logistic regression model consists of four features: open reading frame size, open reading frame coverage, Fickett TESTCODE statistic and hexamer usage bias. CPAT is highly accurate and much faster than other coding potential identification tools. The software accepts input sequences in either FASTA- or BED-formatted data files. We used the human coding probability (CP) cutoff of 0.364 (CP > =0.364 indicates coding sequence; CP < 0.364 indicates noncoding sequence) and the mouse coding probability (CP) cutoff of 0.44, per the recommendation by CPAT.

### Identification of protein-coding genes near lncRNAs

We identified protein-coding genes (both human and mouse) that are in close proximity to the identified lncRNA, within the range of 10 kb. The chromosome locations of those genes were extracted and visualized along with the lncRNA genomic positions in Integrated Genome Browser.

### Functional annotation of lncRNA

The lncRNAs were annotated using Blast2go pro software [70]. The Gene Ontology database was used for the identification of biological processes involved in developmental processes. HiCExplorer(v1.8.1) [71] software was employed for the identification of boundary positions based on available Hi-C data. Using these high-resolution TAD boundaries, we identified and annotated genes present within TADs. TADs were determined from a high-resolution Hi-C matrix (20 kb) with the following parameters: binSize 20 kb, minDepth 60,000, maxDepth 12,000, step 20,000, threshold 0.05.

### Histone modifications

Histone datasets for H3k4me2, H4k20me1, H3k4me3, H3k4me1, H3k27me3, H3k36me3, H3k9ac, H3k79me2, H3k9me3, H3k27ac for myoblasts and myotubes (human and mouse samples) were downloaded from the NCBI SRA database [41].

The quality control procedure filtered out reads with poor quality from raw FASTQ files. The processed reads were aligned with the GRCh38 human genome by the STAR aligner. The MM10 mouse genome was used for alignment of the mouse dataset. The processed BAM files generated by the STAR aligner were sorted by coordinates and reheadered using Samtools.(v1.8, [72]).

### Visualization of DE genes/lncRNAs by integrating RNA-Seq and ChipSeq datasets

To explore the regulation/expression of lncRNAs and protein-coding genes involved in histone modification, we used the ngs.plot program [73]. The list of identified lncRNAs was incorporated for all histones in the human and mouse genomes. We classified the TSS region with a flanking region of 3000 bp. The program utilizes both the RNA-Seq dataset and ChipSeq dataset with arguments “ngs.plot.r -G hg38 -R tss -F chipseq,lincRNA -L 3000” and “ngs.plot.r -G hg38 -R tss -F rnaseq,lincRNA -L 3000”. Transcripts with zero expression were also plotted to check the expression of lncRNAs and protein-coding genes.

### Comparative study of lncRNAs identified in the mouse and human genomes

We developed a method to compare the lncRNAs detected in the mouse (mm10 mouse genome) and human (GRCh38) models based on the protein-coding genes near those lncRNAs. These genes were regarded as the reference genes, and transcripts with maximum logCPM values were chosen. The sequences of identified lncRNAs were extracted, and we used a pairwise alignment strategy to detect sequence variation among mouse and human lncRNAs involved in developmental processes.

### Gene enrichment analysis

The list of upregulated and downregulated genes (logFC +− > = 1, adjP.value < 0.05) was used for gene ontology (GO) enrichment analysis using ClueGO App (Kappa score 0.4, P. Value < 0.05, Bonferroni step down p.value correction) [74]. The analysis was performed in Cytoscape(v 3.7.2, [75]).

### Availability of data and materials

- Mouse transcriptomic dataset: mRNA-seq raw sequence data from Trapnell et al. [35] (GEO Accession ID GSE20846, https://www.ncbi.nlm.nih.gov/geo/query/acc.cgi?acc=GSE20846)) were downloaded from Sequence Read Archive SRA. The dataset SRX017794 (https://www.ncbi.nlm.nih.gov/sra/?term=SRX017794) refers to undifferentiated myoblasts (− 24 h) and SRX017795 (https://www.ncbi.nlm.nih.gov/sra/?term=SRX017795) to the model of differentiated myotubes (60 h). Five samples (2 myoblast and 3 myotube samples) were analysed for the mouse myogenesis model.

GEO accession ID GSE20846: https://www.ncbi.nlm.nih.gov/geo/query/acc.cgi?acc=GSE20846

SRX017794 (undifferentiated myoblasts): https://www.ncbi.nlm.nih.gov/sra/?term=SRX017794

SRX017795 (differentiated myotubes): https://www.ncbi.nlm.nih.gov/sra/?term=SRX017795

b) Human transcriptomic dataset: The human myogenesis raw FASTQ file was downloaded from the GEO database under accession ID GSE79920 (https://www.ncbi.nlm.nih.gov/geo/query/acc.cgi?acc=GSE79920). Zeng et al. [43] performed single-nucleus transcriptome analysis on undifferentiated human KD3 myoblasts and differentiated myotubes as well as mononucleated cells. The myoblasts cells were harvested at 24 h; the myotubes and mononucleated cells were harvested at 72 h after induction of differentiation. A total of 253 samples (133 myoblasts and 120 myotubes) were analysed in our study.

GEO accession ID GSE79920: https://www.ncbi.nlm.nih.gov/geo/query/acc.cgi?acc=GSE79920

c) Reference genome:

Human (GRCh38): https://www.gencodegenes.org/human/

Mouse (mm10): https://www.gencodegenes.org/mouse/

d) Regulatory Elements for Human and Mouse: ENCODE Regulatory Elements database (https://screen.encodeproject.org/).
e) ChIP-seq datasets:

ChIP-seq datasets used for the analysis of human samples were downloaded from the GEO database (GEO Accession ID: GSE19465, https://www.ncbi.nlm.nih.gov/geo/query/acc.cgi?acc=GSE19465).

H3K9me3: https://www.encodeproject.org/experiments/ENCSR503UUB/

H3K9ac: https://www.encodeproject.org/experiments/ENCSR087MJR/

H3K4me3: https://www.encodeproject.org/experiments/ENCSR767NIF/

H3K4me1: https://www.encodeproject.org/experiments/ENCSR823QYQ/

H3K36me3: https://www.encodeproject.org/experiments/ENCSR930OZC/

H3K27me3: https://www.encodeproject.org/experiments/ENCSR454ERY/

H3K27ac: https://www.encodeproject.org/experiments/ENCSR329FXI/

H4K20me1: https://www.encodeproject.org/experiments/ENCSR000AOZ/

H3K79me2: https://www.encodeproject.org/experiments/ENCSR493FIV/

ChIP-seq datasets used for the analysis of mouse samples were downloaded from the GEO database (GEO Accession ID: GSE25308, https://www.ncbi.nlm.nih.gov/geo/query/acc.cgi?acc=GSE25308).

RNApolymerase II myoblast: https://www.ncbi.nlm.nih.gov/geo/query/acc.cgi?acc=GSM721286

RNA polymerase II myotube: https://www.ncbi.nlm.nih.gov/geo/query/acc.cgi?acc=GSM721287

H3K4me1 myoblast: https://www.ncbi.nlm.nih.gov/geo/query/acc.cgi?acc=GSM721288

H3K4me1 myotube: https://www.ncbi.nlm.nih.gov/geo/query/acc.cgi?acc=GSM721289H3K4me2 myoblast: https://www.ncbi.nlm.nih.gov/geo/query/acc.cgi?acc=GSM721290

H3K4me2 myotube: https://www.ncbi.nlm.nih.gov/geo/query/acc.cgi?acc=GSM721291

H3K4me3 myoblast: https://www.ncbi.nlm.nih.gov/geo/query/acc.cgi?acc=GSM721292

H3Kme3 myotube: https://www.ncbi.nlm.nih.gov/geo/query/acc.cgi?acc=GSM721293

H3K27me3 myoblast: https://www.ncbi.nlm.nih.gov/geo/query/acc.cgi?acc=GSM721294

H3K27me3 myotube: https://www.ncbi.nlm.nih.gov/geo/query/acc.cgi?acc=GSM721295

H3K36me3 myoblast: https://www.ncbi.nlm.nih.gov/geo/query/acc.cgi?acc=GSM721296

H3K36me3 myotube: https://www.ncbi.nlm.nih.gov/geo/query/acc.cgi?acc=GSM721297

H3K9Ac myoblast: https://www.ncbi.nlm.nih.gov/geo/query/acc.cgi?acc=GSM721300

H3K9Ac myotube: https://www.ncbi.nlm.nih.gov/geo/query/acc.cgi?acc=GSM721301

H3K18Ac myoblast: https://www.ncbi.nlm.nih.gov/geo/query/acc.cgi?acc=GSM721302

H3K18Ac myotube: https://www.ncbi.nlm.nih.gov/geo/query/acc.cgi?acc=GSM721303

H4K12Ac myoblast: https://www.ncbi.nlm.nih.gov/geo/query/acc.cgi?acc=GSM721304

H4K12Ac myotube: https://www.ncbi.nlm.nih.gov/geo/query/acc.cgi?acc=GSM721305

## Supplementary Information

**Additional file 1: Table S1.** Conserved lncRNAs in Human and Mouse genome along with their positions and functional annotations based on FANTOM database and 3D architecture.

**Additional file 2: Figure S1.** (a) and (b) Expression pattern of lncRNAs in myoblast and myotube. (c) and (d) Expression pattern of nearby genes from Zeng et al. data. Green and orange lines indicate myoblast-specific and myotube-specific lncRNAs in Fig.S1a and Fig.S1.b; nearby genes in Fig.S1c and Fig.S1d. Total number of 500 nearby genes taken into consideration.

**Additional file 3: Figure S2.** Conservation pattern of lncRNAin mouse and human genome. Nucleotide BLAST was performed for sequence alignment of the lncRNAs. X-axis: Human Genome, Y-axis: Mouse Genome.

**Additional file 4: Table S2.** The mean phastcons score, region of conversation and Structure Ensemble Conservation Index (SECI) Score of identified lncRNA.

**Additional file 5: Figure S3.** Structurally conserved region of lncRNA.

**Additional file 6: Figure S4.** Overlap of regulatory elements with conserved lncRNAs in mouse and human datasets.

**Additional file 7.** Complete lists of genomic position of lncRNAs with each of the regulatory elements for human and mouse datasets.

**Additional file 8: Figure S5.** Distribution of H3K9me3,H3K27me3, and H3K36 across lncRNAs and nearby genes. (a) and (b) Distribution of H3K9me3 in lncRNAs and nearby genes. (c) and (d) Distribution of H3K27me3 in lncRNAs and nearby genes. (e) and (f) Distribution of H3K36me3 in lncRNAs and nearby genes. Green and orange lines indicate myoblast-specific and myotube-specific lncRNAs in Fig.S5a,Fig.S5.c, Fig.S5.e; nearby genes in Fig.S5b,Fig.S5d, Fig.S5f. Total number of 500 nearby genes taken into consideration. The indigo lines shows the non-expressing genes.

**Additional file 9: Figure S6.** Distribution of H3K4me1, H3K4me3, H3K9ac, H3K27ac across lncRNAs and nearby genes. (a) and (b) Distribution of H3K4me1 in lncRNAs and nearby genes. (c) and (d) Distribution of H3K4me3 in lncRNAs and nearby genes. (e) and (f) Distribution of H3K9ac in lncRNAs and nearby genes. (g) and (h) Distribution of H327ac in lncRNAs and nearby genes Green and orange lines indicate myoblast-specific and myotube-specific lncRNAs in Fig.S6a, Fig.S6.c, Fig.S6.e, Fig.S6.g; nearby genes in Fig.S6b, Fig.S6d, Fig.S6f, Fig.S6h. Total number of 500 nearby genes taken into consideration. The indigo lines shows the non-expressing genes.

**Additional file 10: Figure S7.** The heatmap of the occurrence of the lncRNAs in histone marks.

**Additional file 11.** The position of lncRNAs along with each histone peaks for both human and mouse genome.

**Additional file 12: Figure S8.** (a) Expression levels of Myf5 by using three different primers Myf5–1, Myf5–2 and Myf5–3 (b) Expression levels of MyoG by using three different primers MyoG-1, MyoG-2 and MyoG-3 during myoblasts culture with 10% FBS, as well as differentiating myotubes at 2, 5 and 7 days after serum starvation.

**Additional file 13: Figure S9.** Melting peaks of PCR product using SYBR Green real-time RT PCR. Quantitative RT-PCR product melting curve of all the six primers to show the specificity and unique PCR product. GAPDH andβ-Actin used as loading control and melting curve of 53–1, 53–2, 53–3, Rik-1, Rik-2 and Rik-3 lncRNA target primers.

**Additional file 14.** Gene ontology results of the lncRNAs and associated genes based on TADs.

**Additional file 15.** Gene enrichment analysis of associated genes involved pathways. (XLS 961 kb)

**Additional file 16: Table S3.** Expression levels of lncRNA Gm28653 measured by using three different primers 53–1, 53–2 and 53–3. Expression level lncRNA 2310043M15Rik measured by using three different primers Rik-1, Rik-2 and Rik-3.

## Data Availability

All data generated or analyzed during this study are included in this published article and its supplementary information files.

## Abbreviations

ncRNA: Non-coding RNA
lncRNA: Long non-coding RNA
CRS: Conserved-RNA Structure
RNA-seq: RNA sequencing
FDR: False discovery rate
logCPM: Log counts per million
PolII: RNA polymerase II TSS Transcription start site
KD3 cell line: Hu5/KD3 cells
HDAC: Histone deacetylase
HAT: Histone acetyltransferases
RT-PCR: Reverse transcriptase-polymerase chain reaction BSA Bovine serum albumin FBS Fetal bovine serum TAD Topologically associated domain
mESC: Mouse embryonic stem cell
hESC: Human embryonic stem cells
IMR90: The human diploid fibroblast strain
ChIP-seq: Chromatin immunoprecipitation followed by sequencing GRCh38 Genome Reference Consortium Human Build 38 MM10 Genome Reference Consortium Mouse Build 38
RSEM: RNA-Seq by Expectation Maximization
EM: Expectation-maximization
ML: Maximum likelihood
TMM: Trimmed mean of M values DMEM Dulbecco’s modified Eagle’s medium DE Differentially expressed
GTF: Gene transfer format CPAT Coding Potential Assessment Tool CP Coding probability BAM Binary Alignment Map
GO: Gene ontology
