# Supplementary material for: Identification of the conserved long non-coding RNAs in myogenesis
Source: BMC Genomics. 2021 May 10;22:336. doi: 10.1186/s12864-021-07615-0 (PMC8112034; doi:10.1186/s12864-021-07615-0)
Supplement: Supplementary file 1 — Additional file 1: Table S1. Conserved lncRNAs in Human and Mouse genome along with their positions and functional annotations based on FANTOM database and 3D architecture. [file 12864_2021_7615_MOESM1_ESM.pdf]

**MouselncRNA**	**Mouse lncRNALocation**	**HumanlncRNA**	**Human lncRNALocation**	**Type of RNA**	**Function from TAD GO**	**Reference**
C130080G10Rik	*chr2:114056313-114064868*	*RP11-814P5.1*	*chr15:34755084-34812923*	p-lncRNA	Differentiation and muscle-development.	
5033430I15Rik	*chr13:45965351-45966177*	*RP1-151F17.2*	*chr6:16764346-16766883*	Probable eRNA	Differentiation and muscle development.	
Gm14635	*chrX:12340637-12345222*	*RP11-320G24.1*	*chrX:40262917-40287720*	e-lncRNA	Developmental process and chromosom/chromatin organization.	
Gm11816	*chr4:9285827-9287333*	*AC022182.1*	*chr8:60918981-60966557*	probable eRNA	Developmental process	
2310039L15Rik	*chr10:95336276-95363216*	*RP11-887P2.5*	*chr12:93714535-93735730*	e-lncRNA	Differentiation, developmental process and chromosom/chromatin organization.	
Gm29237	*chr11:30106394-30108120*	*AC093110.3*	*chr2:54661051-54680045*	probable eRNA	Cell cycle process	
Gm8883	*chr1:71888503-71890587*	*LINC00607*	*chr2:215611563-215843536*	probable eRNA	Differentiation and development process.	

Neat1	*chr19:5843681-5845259*	*NEAT1*	*chr11:65422798-65445540*	p-lncRNA	Developmental process and chromosom/chromatin organization.	(Sunwoo et al. 2009; Neguembor et al. 2014)
<u>*Gm26982*</u>	*chr6:113714971-113716121*	*GHRLOS*	*chr3:10285754-10293449*	probable eRNA	Differentiation, muscle development and chromatin/chromosome organization.	
<u>*Gm5577*</u>	*chr6:87981683-88005471*	*RP13-685P2.7*	*chr3:129345411-129346164*	probable eRNA	Differentiation, developmental process and chromatin/chromosome organization.	
<u>*Gm43878*</u>	*chr6:82831438-82837385*	*RP11-259N19.1*	*chr2:74832655-74833987*	p-lncRNA	Differentiation and developmental process.	
9230105E05Rik	*chr10:120389523-120392793*	*RP11-366L20.2*	*chr12:65855947-65882041*	e-lncRNA	Differentiation and developmental process and chromosom/chromatin organization.	
9230105E05Rik	*chr10:120389523-120392793*	*RP11-221N13.3*	*chr12:65602878-65612997*	e-lncRNA	Differentiation and developmental process and chromosome/chromatin	

					organization.	
<u>*Gm17092*</u>	*chr5:99977686-99978858*	*RP11-127B20.3*	*chr4:82374402-82384027*	p-lncRNA	Developmental process and chromosome/chromatin organization.	
<u>*Gm28653*</u>	*chr1:20679023-20684298*	*LINCMD1*	*chr6:52146814-52151119*	probable eRNA	Differentiation and developmental process and chromosome organization.	(Cesana et al. 2011; Legnini et al. 2014; Neguembor et al. 2014)
<u>*Bvht*</u>	*chr18:61639653-61647472*	*CARMN*	*chr5:149406880-149432835*	probable eRNA	Developmental process	
<u>*Gm20342*</u>	*chr1:63117118-63126837*	*AC007383.3*	*chr2:206084605-206086564*	p-lncRNA	Carbohydrate metabolism	
Crnde	*chr8:92346932-92355869*	*CRNDE*	*chr16:54918870-54920616*	p-lncRNA	Differentiation and developmental process.	
<u>*Junos*</u>	*chr4:95123995-*	*RP4-794H19.1*	*chr1:58882868-*	e-lncRNA	Muscle cell proliferation,	

	95167318		*58896665*		Differentiation and development.	
<u>*Gm13834*</u>	*chr6:31087609-31087912*	*AC058791.1*	*chr7:130876809-130913310*	probable eRNA	no gene	
<u>*AU020206*</u>	*chr7:75769762-75782099*	*RP11-815J21.4*	*chr15:85619623-85670948*	probable eRNA	NA	
C130071C03Rik	*chr13:83738863-83884194*	*MEF2C-AS1*	*chr5:88883399-88944993*	p-lncRNA	No gene	
Mkln1os	*chr6:31384684-31398759*	*LINC-PINT*	*chr7:130945610-131107979*	probable eRNA	Differentiation and developmental process.	
H19	*chr7:142576507-142578095*	*H19*	*chr11:1995176-1996191*	e-lncRNA	Differentiation, muscle development and chromatin/chromosome organization.	(Qin et al. 2017)
Mir155hg	*chr16:84713023-84715245*	*LINC00158*	*chr21:25385820-25431701*	probable eRNA	Differentiation and developmental process.	(Goncalves and Armand 2017)
Mir155hg	*chr16:84713023-84715245*	*MIR155HG*	*chr21:25561909-25575168*	p-lncRNA	Differentiation and developmental process.	(Goncalves and Armand

						2017)
Plet1os	*chr9:50488799-50504805*	*RP11-356J5.12*	*chr11:112290201-112292721*	p-lncRNA	Differentiation, muscle development and chromosome organization.	
Sap30bpos	*chr11:115946450-115952105*	*RP11-474I11.7*	*chr17:75683548-75684799*	probable eRNA	Differentiation, development and chromatin/chromosome organization.	
Sap30bpos	*chr11:115946450-115952105*	*RP11-474I11.8*	*chr17:75679474-75679967*	probable eRNA	Differentiation,development and chromatin/chromosome organization.	
Gm29243	*chr8:34216859-34226145*	*RP11-94H18.1*	*chr8:29815004-29854543*	e-lncRNA	Dephosphorylation	(Guan and Butch 1995)
Malat1	*chr19:5801943-5802640*	*MALAT1*	*chr11:65497762-65506469*	p-lncRNA	Developmental process and chromosome/chromatin organization.	(Chen et al. 2017)
Gm15867	*chr1:192854923-192856246*	*SERTAD4-AS1*	*chr1:210231456-210232972*	p-lncRNA	Differentiation, developmental process and chromatin/chromosome organization.	
3300005D01Rik	*chr17:5799652-5802894*	*RP11-52J3.3*	*chr6:157829143-157830573*	e-lncRNA	Developmental process	
Gm20324	*chr15:82899080-*	*LINC01315*	*chr22:42364525-*	probable eRNA	Differentiation and developmental	

	82905818		*42369236*		process.	
2700038G22Rik	*chr5:23850601-23852979*	*RPL12P10*	*chr7:22881893-22882390*	probable eRNA	Differentiation, development and chromatin/chromosome organization	
2700038G22Rik	*chr5:23850601-23852979*	*AC005682.5*	*chr7:22856528-22861579*	p-lncRNA	Differentiation, development and chromatin/chromosome organization.	
Gm16364	*chr10:39556071-39558062*	*TRAF3IP2-AS1*	*chr6:111565251-111576877*	p-lncRNA	DNA-dependent DNA replication positive regulation of defense response to virus by host intracellular signal transduction	(Ryzhakov et al. 2012)
Lrrc75aos2	*chr11:62605767-62607023*	*LRRC75A-AS1*	*chr17:16438987-16442028*	probable eRNA	Transcription, cation transport	
Lrrc75aos2	*chr11:62605767-62607023*	*RP11-138I1.3*	*chr17:16440479-16440952*	e-lncRNA	Transcription, cation transport	
9330102E08Rik	*chr6:128169704-128183803*	*RP11-253E3.3*	*chr12:3041437-3044950*	probable eRNA	Differentiation and developmental process.	
D130051D11Rik	*chr15:85639747-85645934*	*CITF22-92A6.1*	*chr22:46013606-46015498*	p-lncRNA	No gene	

2310015A10Rik	*chr12:80120548-80132844*	*RPL12P7*	*chr14:68693090-68693583*	probable eRNA	Differentiation and muscle structure development	
5730419F03Rik	*chr1:78595031-78609508*	*AC097461.4*	*chr2:222917387-222919363*	lncRNA	Differentiation and developmental process.	
5730419F03Rik	*chr1:78595031-78609508*	*RP11-525G12.1*	*chr2:222945930-222946388*	lncRNA	Differentiation and developmental process.	
RP24-267C3.3	*chr7:90887072-90940052*	*RP11-851M3.1*	*chr11:84639993-84640565*	lncRNA	Differentiation and developmental process.	
Gm15475	*chr6:84093772-84107007*	*ZNF638-IT1*	*chr2:71373938-71376320*	lncRNA	Differentiation and muscle-development	
Foxo6os	*chr4:120291350-120303892*	*RP11-399E6.4*	*chr1:41375004-41375669*	lncRNA	Differentiation and development	
Bach2os	*chr4:32565639-32571708*	*RP11-63K6.4*	*chr6:89876544-89876878*	lncRNA	Developmental process and chromosome/chromatin organization	
A330074K22Rik	*chr8:120204434-120228230*	*LINC00311*	*chr16:85282958-85285963*	lncRNA	Developmental process	
Srrm4os	*chr5:116438720-*	*RP11-64B16.2*	*chr12:119194850-*	lncRNA	Differentiation, developmental	

	116465487		*119195394*		process and chromosome/chromatin organization.	
1810010H24Rik	*chr11:107028223-107030443*	*RP11-147L13.12*	*chr17:68131462-68131907*	lncRNA	Developmental process	
1810010H24Rik	*chr11:107028223-107030443*	*AC145343.2*	*chr17:68096046-68101474*	lncRNA	Developmental process	
Gm21747	*chr18:10781723-10785883*	*RP11-268I9.1*	*chr18:21854640-21854987*	lncRNA	Differentiation, development and chromatin/chromosome organization.	
Gm17518	*chr16:90727512-90729120*	*AP000265.1*	*chr21:32259804-32261585*	lncRNA	Differentiation, development and chromatin/chromosome organization.	
Gm28231	*chr9:88259895-88263035*	*RP11-30P6.6*	*chr6:85387219-85390186*	lncRNA	Developmental process	
Mirt1	*chr19:53451641-53454094*	*RP11-549L6.2*	*chr10:110257064-110257843*	lncRNA	Dephosphorylation<br>protein import into nucleus	(Guan and Butch 1995; Jeong et al. 2014)
Gm43672	*chr3:22074412-*	*MTND5P15*	*chr3:177010719-*	lncRNA	Differentiation, development and	

	22076142		*177011067*		chromatin/chromosome organization.	

Conserved lncRNAs in Human and Mouse genome along with their positions and functional annotations based on 3D architecture. The lncRNA annotation based on the FANTOM database. p-lncRNA: promoter lncRNA, e-lncRNA: enhancer, probable eRNA: predicted by the expression of H3K27ac. The underlined lncRNAs are not discovered in any other tissues in NONCODE database.

References:

Cesana M, Cacchiarelli D, Legnini I, Santini T, Sthandier O, Chinappi M, Tramontano A, Bozzoni I. 2011. A long noncoding RNA controls muscle differentiation by functioning as a competing endogenous RNA. *Cell* **147**: 358-369.

Chen X, He L, Zhao Y, Li Y, Zhang S, Sun K, So K, Chen F, Zhou L, Lu L et al. 2017. Malat1 regulates myogenic differentiation and muscle regeneration through modulating MyoD transcriptional activity. *Cell Discov* **3**: 17002.

Goncalves TJM, Armand AS. 2017. Non-coding RNAs in skeletal muscle regeneration. *Noncoding RNA Res* **2**: 56-67.

Guan KL, Butch E. 1995. Isolation and characterization of a novel dual specific phosphatase, HVH2, which selectively dephosphorylates the mitogen-activated protein kinase. *The Journal of biological chemistry* **270**: 7197-7203.

Jeong DG, Wei CH, Ku B, Jeon TJ, Chien PN, Kim JK, Park SY, Hwang HS, Ryu SY, Park H et al. 2014. The family-wide structure and function of human dual-specificity protein phosphatases. *Acta crystallographica Section D, Biological crystallography* **70**: 421-435.

Legnini I, Morlando M, Mangiavacchi A, Fatica A, Bozzoni I. 2014. A feedforward regulatory loop between HuR and the long noncoding RNA linc-MD1 controls early phases of myogenesis. *Molecular cell* **53**: 506-514.

Neguembor MV, Jothi M, Gabellini D. 2014. Long noncoding RNAs, emerging players in muscle differentiation and disease. *Skelet Muscle* **4**: 8.

Qin CY, Cai H, Qing HR, Li L, Zhang HP. 2017. Recent advances on the role of long non-coding RNA H19 in regulating mammalian muscle growth and development. *Yi chuan = Hereditas* **39**: 1150-1157.

Ryzhakov G, Blazek K, Lai CC, Udalova IA. 2012. IL-17 receptor adaptor protein Act1/CIKS plays an evolutionarily conserved role in antiviral signaling. *Journal of immunology (Baltimore, Md : 1950)* **189**: 4852-4858.

Sunwoo H, Dinger ME, Wilusz JE, Amaral PP, Mattick JS, Spector DL. 2009. MEN epsilon/beta nuclear-retained non-coding RNAs are up-regulated upon muscle differentiation and are essential components of paraspeckles. *Genome research* **19**: 347-359.
