# Supplementary figures and images for: Identification of the conserved long non-coding RNAs in myogenesis
Source: BMC Genomics. 2021 May 10;22:336. doi: 10.1186/s12864-021-07615-0 (PMC8112034; doi:10.1186/s12864-021-07615-0)

Mouse

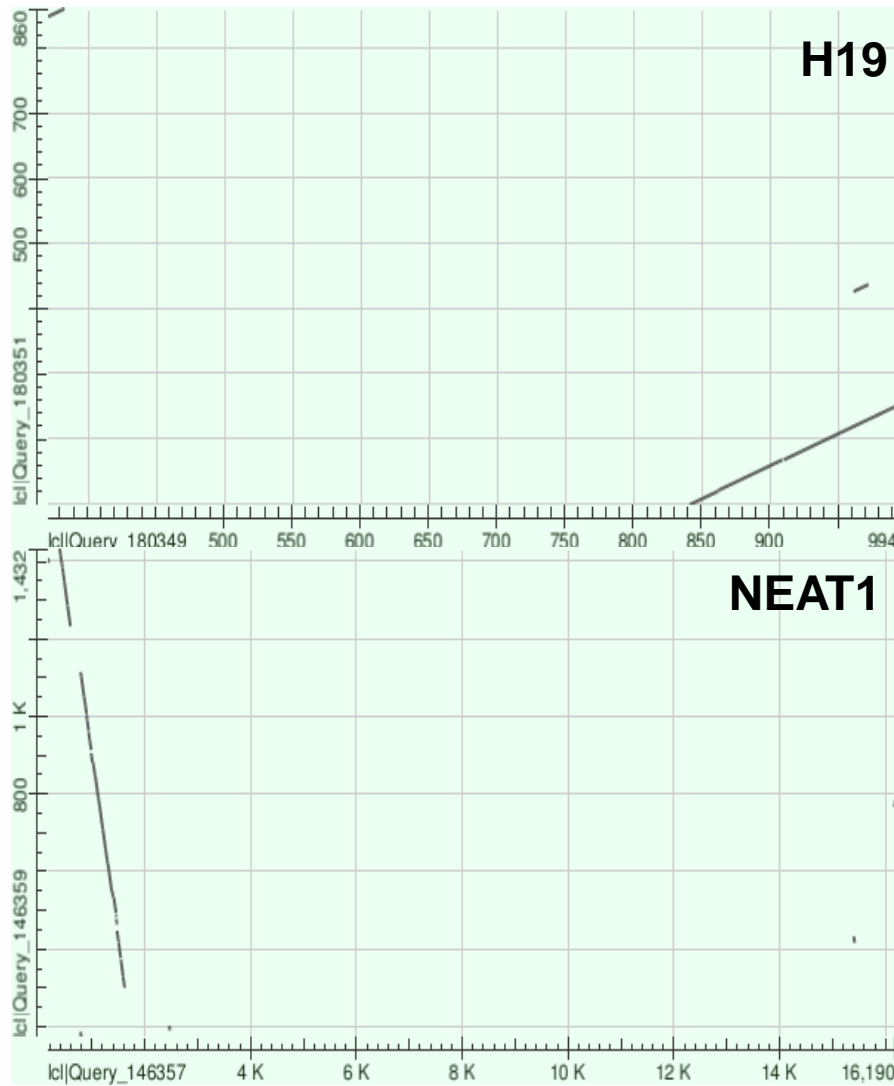

Human

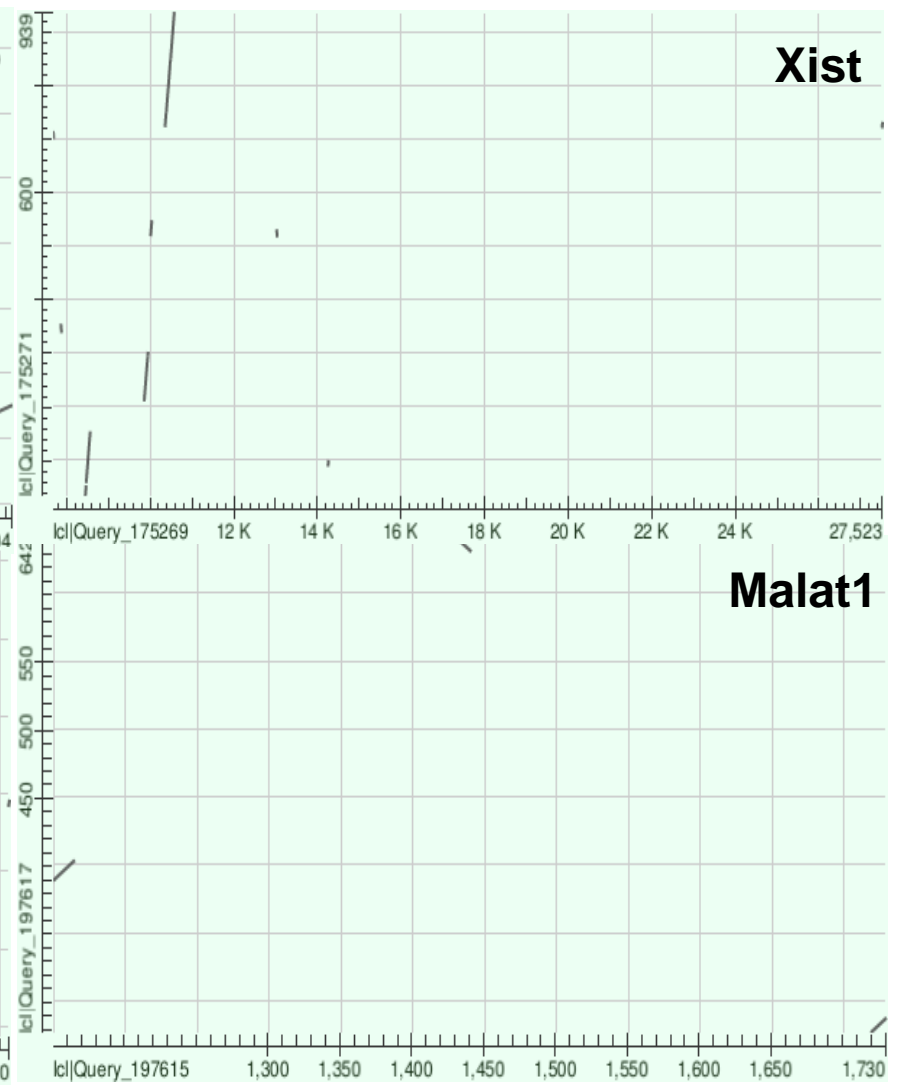

Supplement: Supplementary file 3 — Additional file 3: Figure S2. Conservation pattern of lncRNAin mouse and human genome. Nucleotide BLAST was performed for sequence alignment of the lncRNAs. X-axis: Human Genome, Y-axis: Mouse Genome. [file 12864_2021_7615_MOESM3_ESM.pdf]

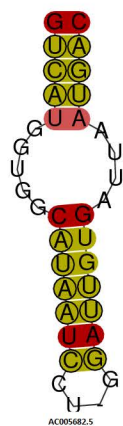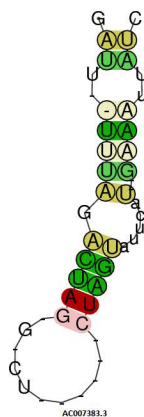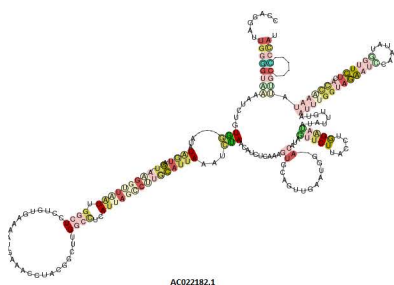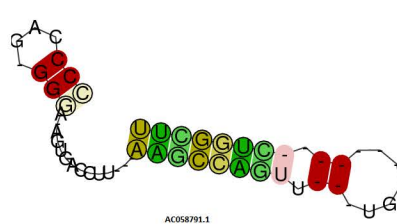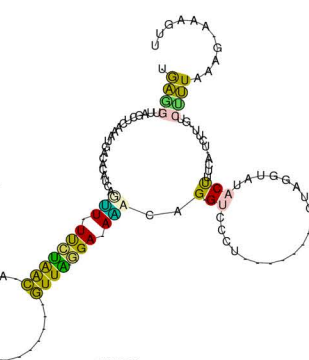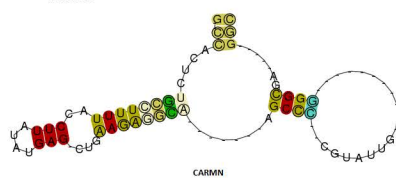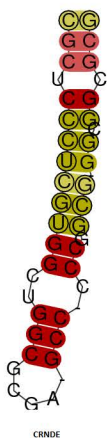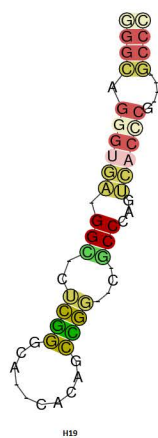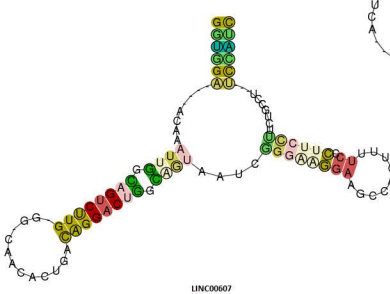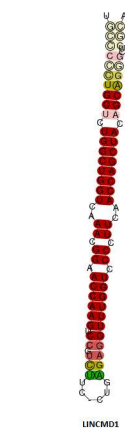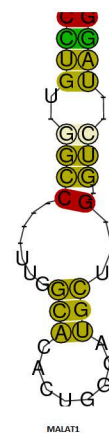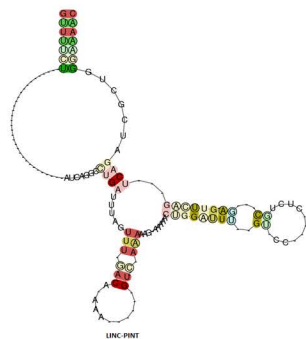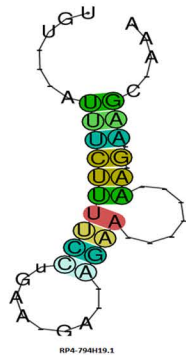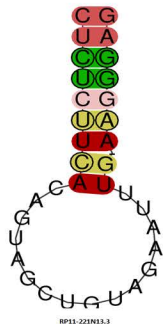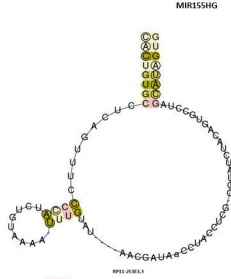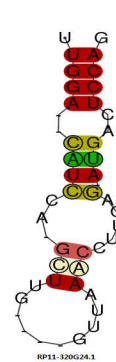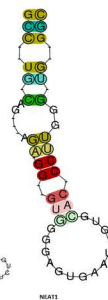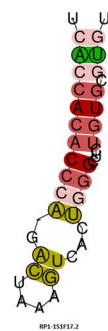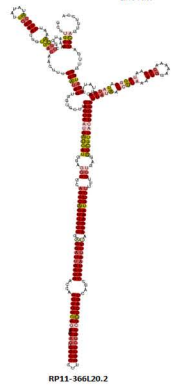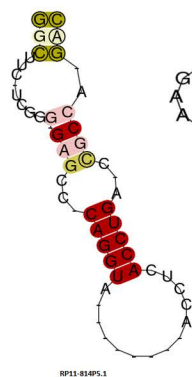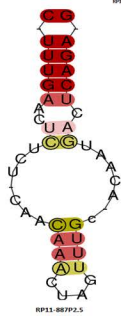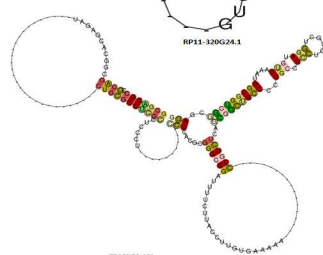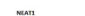

Supplement: Supplementary file 5 — Additional file 5: Figure S3. Structurally conserved region of lncRNA. [file 12864_2021_7615_MOESM5_ESM.pdf]

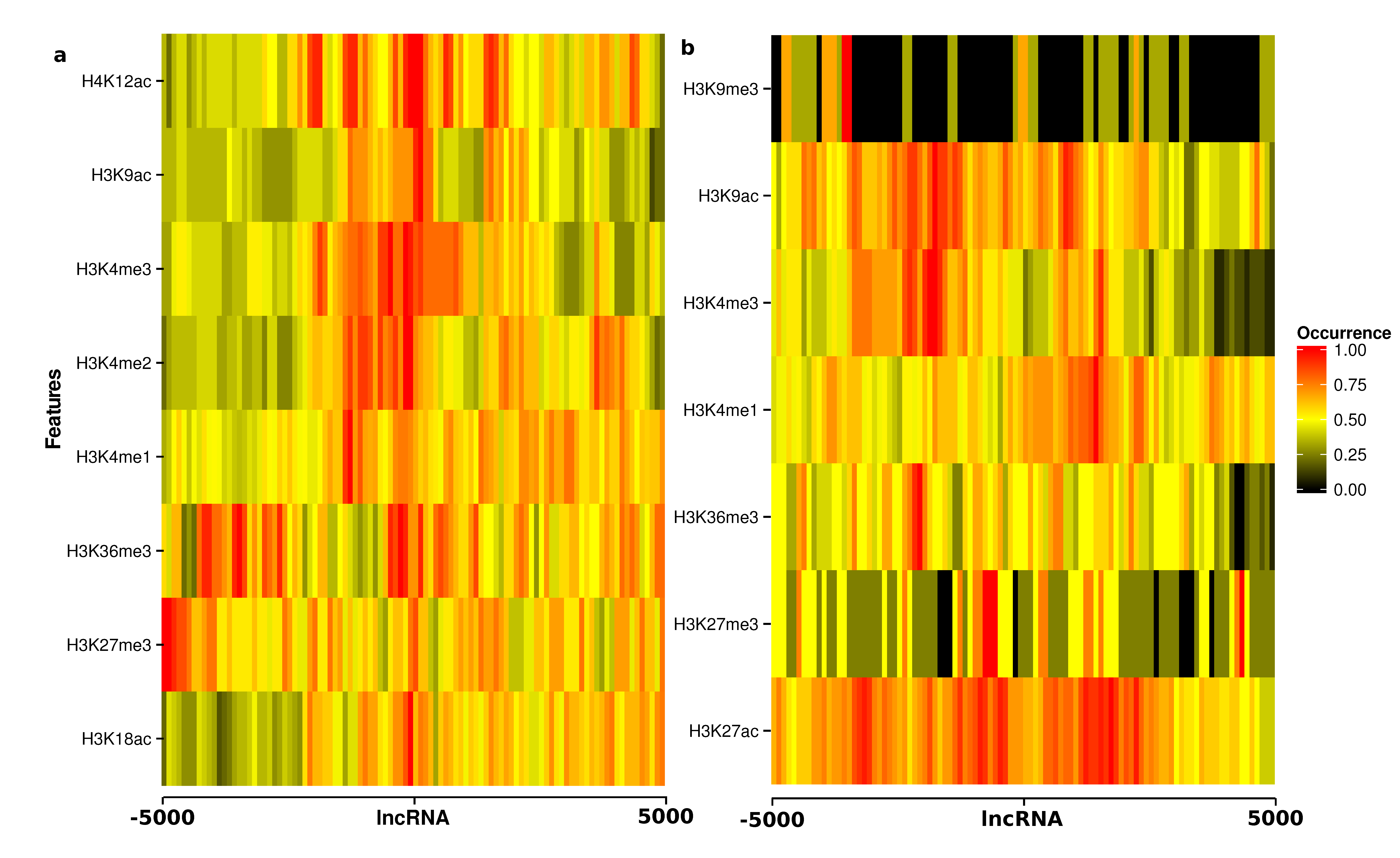

Supplement: Supplementary file 10 — Additional file 10: Figure S7. The heatmap of the occurrence of the lncRNAs in histone marks. [file 12864_2021_7615_MOESM10_ESM.tiff]

## GAPDH

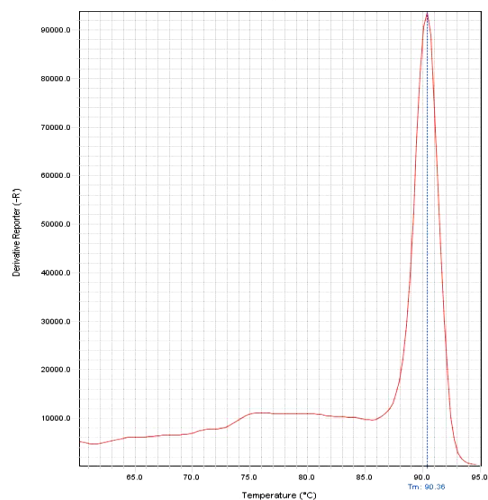

## $\beta$ -ACTIN

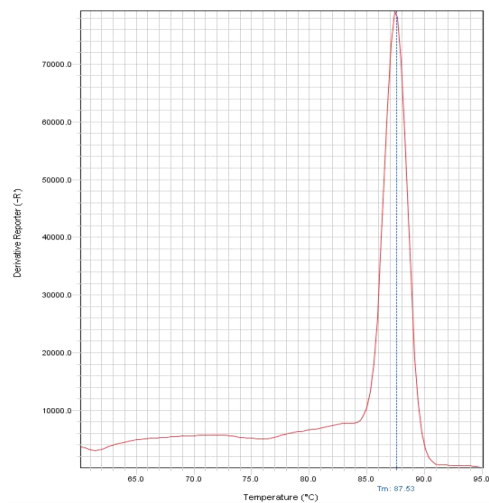

## 53-1

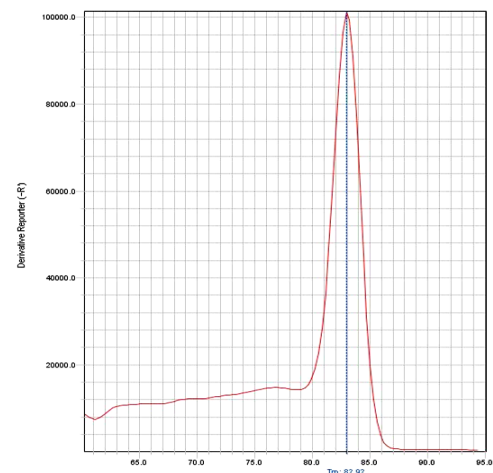

## 53-2

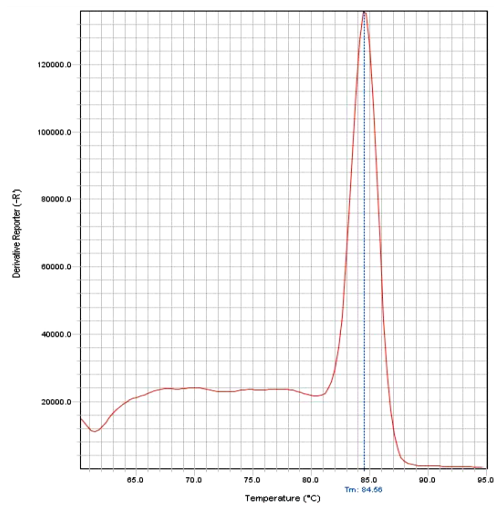

## 53-3

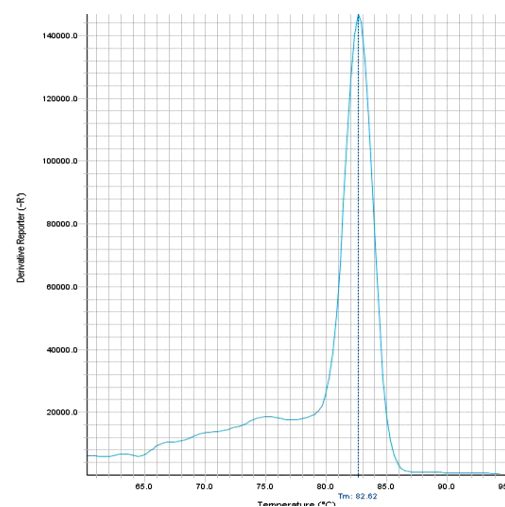

## Rik-1

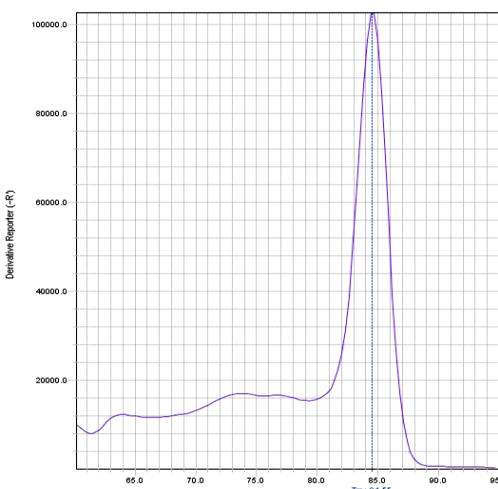

## Rik-2

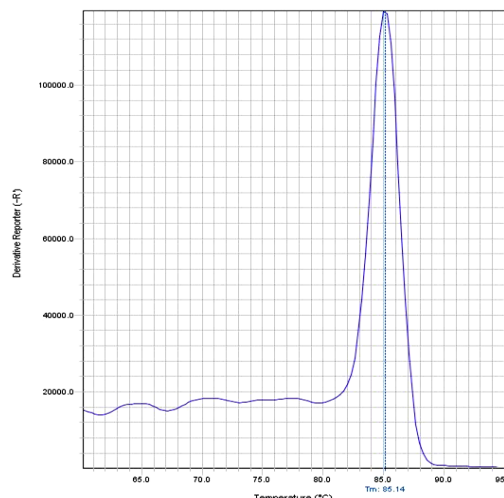

## Rik-3

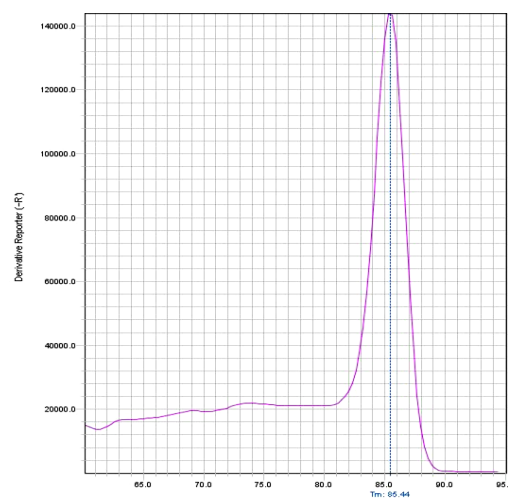

Supplement: Supplementary file 13 — Additional file 13: Figure S9. Melting peaks of PCR product using SYBR Green real-time RT PCR. Quantitative RT-PCR product melting curve of all the six primers to show the specificity and unique PCR product. GAPDH andβ-Actin used as loading control and melting curve of 53–1, 53–2, 53–3, Rik-1, Rik-2 and Rik-3 lncRNA target primers. [file 12864_2021_7615_MOESM13_ESM.pdf]
