# Supplementary material for: Identification of the conserved long non-coding RNAs in myogenesis
Source: BMC Genomics. 2021 May 10;22:336. doi: 10.1186/s12864-021-07615-0 (PMC8112034; doi:10.1186/s12864-021-07615-0)
Supplement: Supplementary file 4 — Additional file 4: Table S2. The mean phastcons score, region of conversation and Structure Ensemble Conservation Index (SECI) Score of identified lncRNA. [file 12864_2021_7615_MOESM4_ESM.pdf]

HumanlncRNA	Mean phastcon score	Region of Conservation	Structure Ensemble Conservation Index(SECI) Score
RP11-814P5.1	0.5346	120-161	62.69
RP1-151F17.2	0.2967	1992-2031	64.4
RP11-320G24.1	0.1045	453-491	67.34
AC022182.1	0.0868	12-207	67.74
RP11-887P2.5	0.8302	1039-1079	68.31
AC093110.3	NA	NA	NA
LINC00607	0.029	55-144	64.58
NEAT1	0.266,0.4508	1062-1105, 1483-1520	61,96,64.82
GHRLOS	NA	NA	NA
RP13-685P2.7	NA	NA	NA
RP11-259N19.1	NA	NA	NA
RP11-366L20.2	0.9828	1883-2146	71.67
RP11-221N13.3	0.5953	107-140	60.37
RP11-127B20.3	NA	NA	NA
LINCMD1	0.9995	71-157	69.12
CARMN	0.8526,1.0000,0.0362	1-29,50-93,291-368	60.86,69.80,54.16
AC007383.3	0.2705,0.9620	316-352, 687-747	57.32, 70.92
CRNDE	0.2229, 0.1698	242-280, 389-429	58.51,56.34
RP4-794H19.1	0.0886	1_13	60.5
AC058791.1	0.3042	1908-1944	55.07
RP11-815J21.4	NA	NA	NA
MEF2C-AS1	NA	NA	NA
LINC-PINT	0.1680, 0.1918, 0.3583	1-9, 229-319, 836-885	64.52, 68.52, 55.34
H19	0.0807, 0.1389,0.1305,0.1231	431-479, 509-589, 808-890, 1021-1121	49.81, 70.31,68.58,59.13
LINC00158	NA	NA	NA
MIR155HG	0.8864	77-235	68.55
RP11-356J5.12	NA	NA	NA
RP11-474I11.7	NA	NA	NA
RP11-474I11.8	NA	NA	NA
RP11-94H18.1	NA	NA	NA
MALAT1	0.3704, 0.8749, 0.9419,0.0023	2977-3011, 3806-3868, 5388-5458, 5766-5800	56.00, 64.52,66.65,59.54
SERTAD4-AS1	NA	NA	NA
RP11-52J3.3	NA	NA	NA
RPL12P10	NA	NA	NA
AC005682.5	0.0042	321-356	58.65
TRAF3IP2-AS1	0.2637	127-258	66.3
LRRC75A-AS1	NA	NA	NA
RP11-138I1.3	NA	NA	NA
RP11-253E3.3	0.4947,0.0006	555-633,1527-1590	70.07,55.81

CITF22-92A6.1	NA	NA	NA
RPL12P7	NA	NA	NA
AC097461.4	0.0003	1_83	67.69
RP11-525G12.1	NA	NA	NA
RP11-851M3.1	NA	NA	NA
ZNF638-IT1	NA	NA	NA
RP11-399E6.4	NA	NA	NA
RP11-63K6.4	NA	NA	NA
LINC00311	NA	NA	NA
RP11-64B16.2	NA	NA	NA
RP11-147L13.12	NA	NA	NA
AC145343.2	NA	NA	NA
RP11-268I9.1	NA	NA	NA
AP000265.1	NA	NA	NA
RP11-30P6.6	NA	NA	NA
RP11-549L6.2	NA	NA	NA
MTND5P15	NA	NA	NA

The Mean Phastcons score and region of conversation were annoated using RNA Central Database. The alignment considered 100 vertebrates species. Structure Ensemble Conservation Index(SECI) Score was annotated using Conserved-RNA Structure (CRS) database.
