# Supplementary material for: Identification of the conserved long non-coding RNAs in myogenesis
Source: BMC Genomics. 2021 May 10;22:336. doi: 10.1186/s12864-021-07615-0 (PMC8112034; doi:10.1186/s12864-021-07615-0)
Supplement: Supplementary file 12 — Additional file 12: Figure S8. (a) Expression levels of Myf5 by using three different primers Myf5–1, Myf5–2 and Myf5–3 (b) Expression levels of MyoG by using three different primers MyoG-1, MyoG-2 and MyoG-3 during myoblasts culture with 10% FBS, as well as differentiating myotubes at 2, 5 and 7 days after serum starvation. [file 12864_2021_7615_MOESM12_ESM.pdf]

a

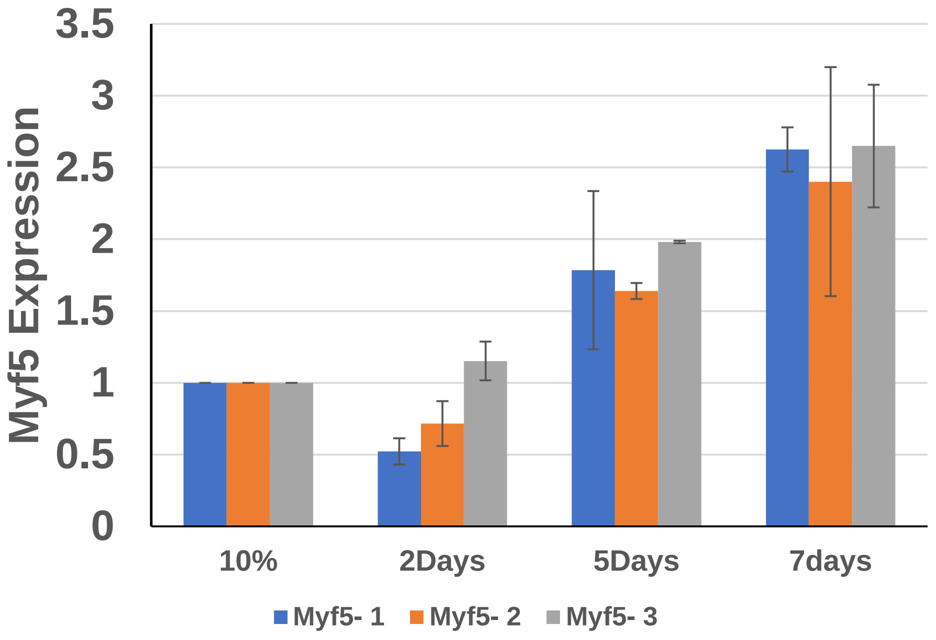

Myf5 Expression
3.5
3
2.5
2
1.5
1
0.5
0
10%
2Days
5Days
7days
Myf5- 1
Myf5- 2
Myf5- 3

b

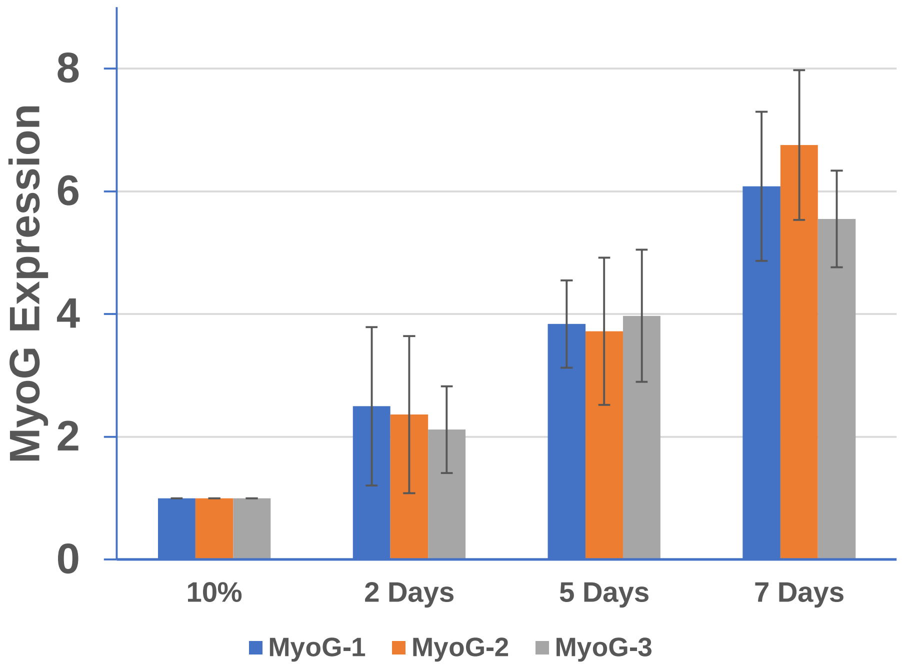

MyoG Expression
8
6
4
2
0
10%
2 Days
5 Days
7 Days
MyoG-1
MyoG-2
MyoG-3

Primer Details:

Myf5 (Myf5-1, Myf5-2 and Myf5-3)

Myf5-1

1)Parameter Set: qPCR Intercalating Dyes (Primers only)
2)Sequence Name: Sequence 1
3)Amplicon Length: 105

	Start	Stop	Length	Tm	GC %
CCTCCAGCTCCAGACTTATCTA(Forward)	937	959	22	62	50
CTTCAGCTTCAGGGCTTCTT(Reverse)	1021	1041	20	62	50

Myf5-2

1)Parameter Set: qPCR Intercalating Dyes (Primers only)
2)Sequence Name: Sequence 1
3)Amplicon Length: 109

	Start	Stop	Length	Tm	GC %
GGGAACAGGTGGAGAACTATTAC(Forward)	619	642	23	62	47.8
CAGACAGGGCTGTTACATTCA (Reverse)	707	728	21	62	47.6

Myf5-3

1)Parameter Set: qPCR Intercalating Dyes (Primers only)
2)Sequence Name: Sequence 1
3)Amplicon Length: 113

	Start	Stop	Length	Tm	GC %
AGACAAGCTGGGCAGAATAC (Forward)	1045	1065	20	62	50
CAGGCAGAGGAGAATCCATTATT(Reverse)	1135	1158	23	62	43.5

MyoG (MyoG-1, MyoG-2 and MyoG-3)

MyoG-1

1)Parameter Set: qPCR Intercalating Dyes (Primers only)
2)Sequence Name: Sequence 1
3)Amplicon Length: 102

	Start	Stop	Length	Tm	GC %
AGAGAAAGATGGAGTCCAGAGA(Forward)	950	972	22	62	45.5
GGGTGGAATTCGAGGCATATTA(Reverse)	1030	1052	22	62	45.5

MyoG-2

1)Parameter Set: qPCR Intercalating Dyes (Primers only)
2)Sequence Name: Sequence 1
3)Amplicon Length: 104

	Start	Stop	Length	Tm	GC %
GTGGGCATGTAAGGTGTGTAA(Forward)	253	274	21	62	47.6
CGAAGGCCTCATTCACTTTCT (Reverse)	336	357	21	62	47.6

MyoG-3

1)Parameter Set: qPCR Intercalating Dyes (Primers only)
2)Sequence Name: Sequence 1
3)Amplicon Length: 93

	Start	Stop	Length	Tm	GC %
CCACAATCTGCACTCCCTTAC (Forward)	637	658	21	63	52.4
TCTCAGTTGGGCATGGTTTC (Reverse)	710	730	20	62	50

Expression levels of Myf5 gene measured by using three different primers Myf5-1, Myf5-2 and Myf5-3. Expression level of MyoG measured by using three different primers MyoG-1, MyoG-2 and MyoG-3.
