# Supplementary material for: Identification of the conserved long non-coding RNAs in myogenesis
Source: BMC Genomics. 2021 May 10;22:336. doi: 10.1186/s12864-021-07615-0 (PMC8112034; doi:10.1186/s12864-021-07615-0)
Supplement: Supplementary file 16 — Additional file 16: Table S3. Expression levels of lncRNA Gm28653 measured by using three different primers 53–1, 53–2 and 53–3. Expression level lncRNA 2310043M15Rik measured by using three different primers Rik-1, Rik-2 and Rik-3. [file 12864_2021_7615_MOESM16_ESM.pdf]

Primer Details:

Gm28653 (53-1, 53-2 and 53-3)

1) Parameter Set: qPCR Intercalating Dyes (Primers only)
2) Sequence Name: Sequence 1
3) Amplicon Length: 98

1) 53-1

	Start	Stop	Length	Tm	GC%
Forward **GACCACTACATCCCTCTTTCAG (Sense)**	899	921	22	62	50
Reverse **CTGTTCCACACTCTCACCTATC (AntiSense)**	975	997	22	62	50

2) 53-2

Parameter Set: qPCR Intercalating Dyes (Primers only)
Sequence Name: Sequence 1
Amplicon Length: 110

	Start	Stop	Length	Tm	GC%
Forward **ATGAGCATGCCACCCTATTC (Sense)**	948	968	20	62	50
Reverse **CGATCCTATGCTCAGTCCTCTA (AntiSense)**	1036	1058	22	62	50

3) 53-3

Parameter Set: qPCR Intercalating Dyes (Primers only)
Sequence Name: Sequence 1
Amplicon Length: 109

	Start	Stop	Length	Tm	GC%
Forward **CTTCTTCGTTCCTTGGCTACA (Sense)**	109	130	21	62	47.6
Reverse **GGGAGCATAGTTGACCTGAAA (AntiSense)**	197	218	21	62	47.6

2310043M15Rik (Rik-1, Rik-2 and Rik-3)

1) Rik-1

Parameter Set: qPCR Intercalating Dyes (Primers only)
Sequence Name: Sequence 1
Amplicon Length: 90

	Start	Stop	Length	Tm	GC%
Forward **CTGATGCTCTTCCGAGGATAAC (Sense)**	227	249	22	62	50

Reverse **GAAGGGAGTGGTGTTAGTGTG (AntiSense)**	296	317	21	62	52.4

2) Rik-2

Parameter Set: qPCR Intercalating Dyes (Primers only)
Sequence Name: Sequence 1
Amplicon Length: 102

	Start	Stop	Length	Tm	GC%
Forward **GCCCAGGAACAGGCTAAA (Sense)**	155	173	18	62	55.6
Reverse **CAGTCTCAGTTATCCTCGGAAG (AntiSense)**	235	257	22	61	50

3) Rik-3

Parameter Set: qPCR Intercalating Dyes (Primers only)
Sequence Name: Sequence 1
Amplicon Length: 127

	Start	Stop	Length	Tm	GC%
Forward **GTATTGTGGCTCTGCTCTCTAC (Sense)**	602	624	22	62	50
Reverse **CAGTGGTGTCCTGTAGTGTTT (AntiSense)**	708	729	21	62	47.6

Expression levels of lncRNA Gm28653 measured by using three different primers 53-1, 53-2 and 53-3. Expression level lncRNA 2310043M15Rik measured by using three different primers Rik-1, Rik-2 and Rik-3.
